# Toward a generalizable machine learning workflow for neurodegenerative disease staging with focus on neurofibrillary tangles

**DOI:** 10.1186/s40478-023-01691-x

**Published:** 2023-12-18

**Authors:** Juan C. Vizcarra, Thomas M. Pearce, Brittany N. Dugger, Michael J. Keiser, Marla Gearing, John F. Crary, Evan J. Kiely, Meaghan Morris, Bartholomew White, Jonathan D. Glass, Kurt Farrell, David A. Gutman

**Affiliations:** 1 The Wallace H. Coulter Department of Biomedical Engineering, Georgia Institute of Technology and Emory University, 313 Ferst Dr NW, Atlanta, GA 30332 USA; 2 Department of Pathology, Division of Neuropathology, University of Pittsburgh Medical Center, Room S701 Scaife Hall 3550 Terrace Street, Pittsburgh, PA 15261 USA; 3 Department of Pathology and Laboratory Medicine, University of California-Davis School of Medicine, 3400A Research Building III Sacramento, Davis, CA 95817 USA; 4 Department of Pharmaceutical Chemistry, Department of Bioengineering and Therapeutic Sciences, Institute for Neurodegenerative Diseases, Kavli Institute for Fundamental Neuroscience, and Bakar Computational Health Sciences Institute, University of California, 675 Nelson Rising Ln, Box 0518, San Francisco, CA 94143 USA; 5 Department of Neurology, Emory University School of Medicine, 12 Executive Park Dr NE, Atlanta, GA 30322 USA; 6 Department of Pathology and Laboratory Medicine, Emory University School of Medicine, 1364 Clifton Rd, Atlanta, GA 30322 USA; 7 Departments of Pathology, Neuroscience, and Artificial Intelligence and Human Health, Icahn School of Medicine at Mount Sinai, New York, NY USA; 8 Neuropathology Brain Bank and Research Core, Friedman Brain Institute, Icahn School of Medicine at Mount Sinai, New York, NY USA; 9 Ronald M. Loeb Center for Alzheimer’s Disease, Icahn School of Medicine at Mount Sinai, New York, NY USA; 10 Department of Pathology, Icahn School of Medicine at Mount Sinai, Icahn Building 9th Floor, Room 20A, 1425 Madison Avenue, New York, NY 10029 USA; 11 Department of Pathology, Johns Hopkins School of Medicine, Baltimore, MD 21218 USA; 12 Department of Pathology, Beth Israel Deaconess Medical Center, 330 Brookline Avenue, Boston, MA 02215 USA; 13 Center for Neurodegenerative Disease, Emory University School of Medicine, Whitehead Biomedical Research Building, 615 Michael Street, 5th Floor, Suite 500, Atlanta, GA 30322 USA; 14 Department of Pathology, Icahn School of Medicine at Mount Sinai, Icahn Building 9th Floor, L9-02C, 1425 Madison, Avenue, New York, NY USA

**Keywords:** Neuropathology, Machine learning, Model-assisted-labeling, Alzheimer’s disease, Neurofibrillary tangles, Braak NFT staging, Whole-slide-images

## Abstract

Machine learning (ML) has increasingly been used to assist and expand current practices in neuropathology. However, generating large imaging datasets with quality labels is challenging in fields which demand high levels of expertise. Further complicating matters is the often seen disagreement between experts in neuropathology-related tasks, both at the case level and at a more granular level. Neurofibrillary tangles (NFTs) are a hallmark pathological feature of Alzheimer disease, and are associated with disease progression which warrants further investigation and granular quantification at a scale not currently accessible in routine human assessment. In this work, we first provide a baseline of annotator/rater agreement for the tasks of Braak NFT staging between experts and NFT detection using both experts and novices in neuropathology. We use a whole-slide-image (WSI) cohort of neuropathology cases from Emory University Hospital immunohistochemically stained for Tau. We develop a workflow for gathering annotations of the early stage formation of NFTs (Pre-NFTs) and mature intracellular (iNFTs) and show ML models can be trained to learn annotator nuances for the task of NFT detection in WSIs. We utilize a model-assisted-labeling approach and demonstrate ML models can be used to aid in labeling large datasets efficiently. We also show these models can be used to extract case-level features, which predict Braak NFT stages comparable to expert human raters, and do so at scale. This study provides a generalizable workflow for various pathology and related fields, and also provides a technique for accomplishing a high-level neuropathology task with limited human annotations.

**Supplementary Information:**

The online version contains supplementary material available at 10.1186/s40478-023-01691-x.

## Introduction

Neuropathologic analysis of brain tissue is fundamental to enhancing our understanding of Alzheimer Disease (AD) and related dementias [1, 2]. This process involves careful review of brain tissue using a variety of stains and antibodies, by experts, which is the current gold standard for diagnosis [3]. In contrast, ante-mortem diagnosis is based on clinical findings, such as neurological symptoms, cognitive test results, family background, including genetic predisposition (e.g. APOE genotype), cerebral spinal fluid biomarkers, and other laboratory and neuroimaging modalities [4–6]. The ante-mortem diagnosis is typically validated against the neuropathology diagnosis to provide a better understanding of the pathology present in the brain and how it relates to clinical symptoms and disease progression [7]. This feedback loop is critical for improving our understanding of these complex diseases, which in turn provides guidance in the development of future diagnostic biomarkers.

Over the last decades there have been significant advances to improve the diagnostic process, including better staining techniques, improvements of diagnostic staging systems, and increased access to large digitized tissue slides known as whole-slide-images (WSI) [2, 6, 8–13]. Additionally, and in order to increase the consistency of neuropathology diagnosis across research centers, standardized qualitative and semi-quantitative staging systems, which rely on visual inspection of the tissue, often by a single expert, have been developed [14–18].

AD neuropathologic changes have classically been defined as abnormal accumulation of amyloid beta (aβ) and tau proteins [19]. Misfolded aβ forms extracellular structures known as aβ plaques, which are hypothesized to hinder communications between neurons and other cells [20, 21]. Abnormally hyperphosphorylated tau can create intraneuronal inclusions known as neurofibrillary tangles (NFTs) which lead to cell death over a prolonged period of time [8, 22]. The current standard for neuropathology diagnosis of AD is set forth by the National Institute on Aging—Alzheimer's Association (NIA-AA) [6, 14, 15, 23–25]. Part of this criteria is the Braak NFT staging system, which is predicated upon the presence or absence of NFTs in select brain regions [26]. Braak NFT stage spans from no (stage 0) or very little NFT pathology (stage I), to abundant NFT pathology throughout the entorhinal, limbic, and isocortical regions of the brain (stage VI) giving a single stage for each case [14, 27].

Semi-quantitative neuropathology schemes display good inter-rater agreement among experts for cases with little or abundant pathology, but fare worse in intermediate stages [28–32]. Disagreements are in part caused by differences in neuropathology evaluation between institutions, and sometimes even between the pathologists/experts within a given institution due to variation in protocols for tissue and slide preparation between laboratories. Our group has also previously demonstrated differences amongst institutions including size of tissue section sampled, antibody used in immunohistochemistry, brain regions collected, and variations in staging protocol used [31, 32]. While semi-quantitative scoring systems remain essential in neuropathology, such systems, in their efforts to simplify and standardize, invariably do not fully capture the complexity of these rich datasets. However, recent breakthroughs in machine learning (ML) and computer vision have had a broad impact across a wide set of disciplines, and may hold some promise for addressing these issues [33, 34].

Within the neuropathology literature, for example, ML has been shown to reliably detect aβ plaques and NFTs [35–39]. Yet progress in this space is hampered by various factors, such as the large amount of variation seen in neuropathology cohorts, pre-analytical variables such as tissue preparation and staining parameters, variations in digital imaging acquisition between scanners, and the need for large, typically expert labeled datasets to train ML models [31, 32, 40]. Current published workflows also involve hours of computational effort per WSI, generally require in depth knowledge of programming, and have no easy method to implement workflows or visualize results at scale [41].

Creating labeled datasets which have adequate size and fidelity for ML is challenging. It demands investment of time and effort from experts (i.e. neuropathologists) who are in high demand [42]. In some contexts, crowd-sourcing approaches using systems like Amazon’s MechanicalTurk can reduce the need for domain experts, depending on the complexity of the task [43–47]. In the medical field this is not always possible however, due to the expertise required. For example, while it may be easy to train non-experts to identify individual cells on a slide, accurately identifying subtypes such as astrocytes or oligodendrocytes can be a much harder task. Furthermore, even experts often disagree with each other, making defining the ground truth needed to train a robust model complicated [30].

In this work, we focus on two tasks: NFT detection and Braak NFT staging [14]. We demonstrate a computational workflow which detects neuropathology-relevant histologic features at scale, and show computational imaging paradigms can be utilized in neuropathology research with high levels of efficacy, while also reducing expert burden. This was achieved using YOLO (You Only Look Once) ML models capable of detecting early stage formation of NFTs, the pre-tangle (Pre-NFT) phase, as well as mature intracellular NFTs (iNFTs) in WSIs. These models were developed in house and refined for this purpose [8]. YOLO models have been shown to be effective in similar neuropathology based tasks, with recent work showing its ability to accurately and reliably detect aβ pathology in WSIs [48]. Koga et al. [49], used an older implementation of YOLO, YOLOv3, to detect five different types of tau inclusions, and used these to successfully differentiate tauopathies in neuropathology cases. In this work, Pre-NFT/iNFT YOLO detection is first used to extract a set of descriptive features for neuropathology cases, which in turn are used to recreate Braak NFT staging comparable to human expert raters. We also assessed Braak NFT stage inter-rater and NFT inter-annotator agreement in our cohorts, and used this as a baseline to evaluate our models. Given the challenges of gathering ground truth labels, we assembled a team of experts, from whom we derive consensus, and later evaluated the impact of including novice annotators. Finally, we developed a facile model-assisted-labeling workflow to further enhance the robustness of our consensus labeled dataset.

## Materials and methods

### Cohorts/datasets

**Fig. 1:**
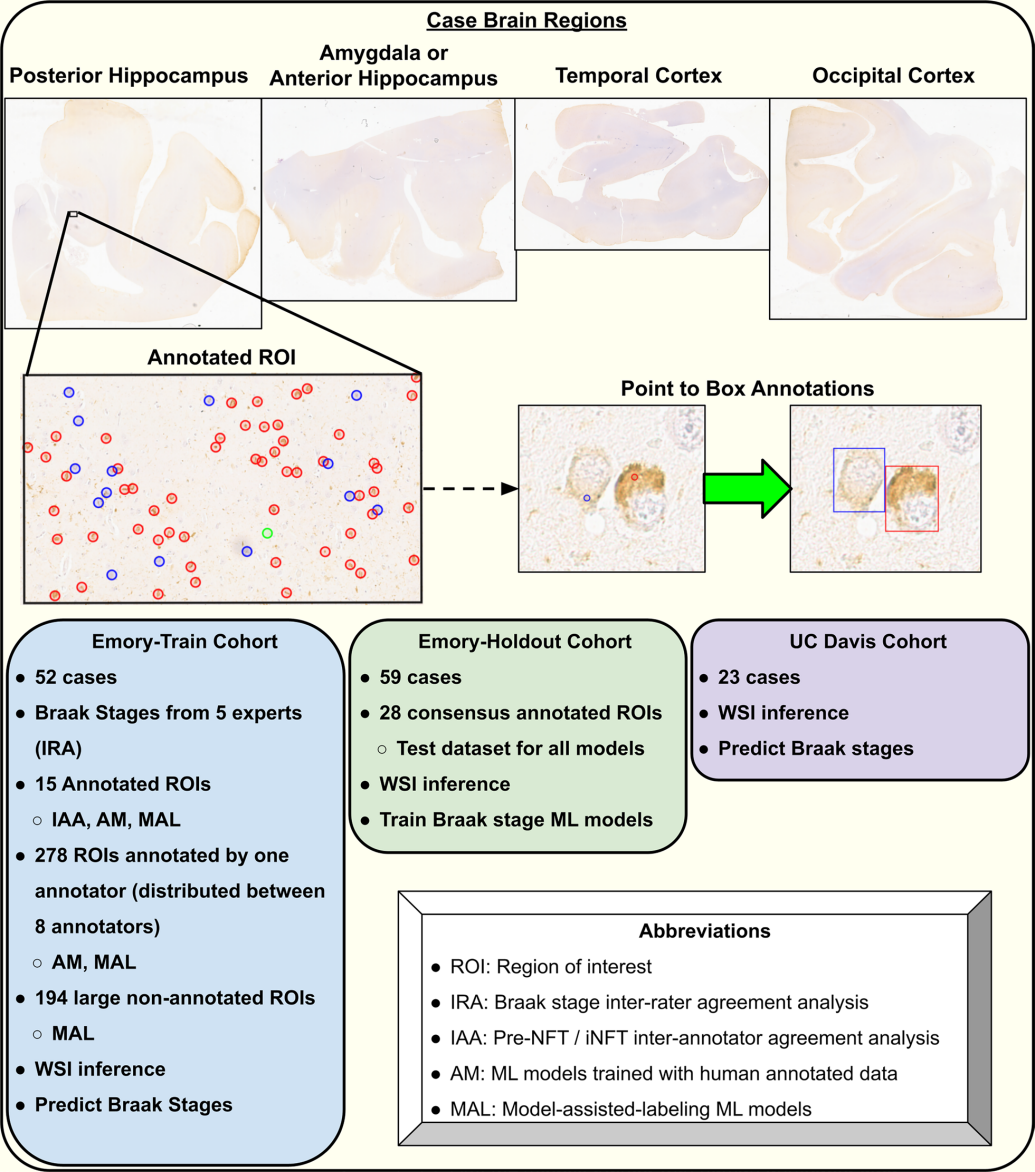
Overview of project data contribution. Each neuropathology case contained four regions immunohistochemically stained for tau pathology (top). All cases included WSIs from the posterior hippocampus, temporal cortex, and occipital cortex. Emory cases included the amygdala while UC Davis cases used the anterior hippocampus instead. WSIs from the Emory-Train cohort included one or more ROIs that were annotated by either multiple annotators for inter-annotator-agreement analysis, a single annotator, or not annotated (middle, left). Not all WSIs contained ROIs in this cohort however. Annotations were collected as single pixels (circles for visualization) and converted to box annotations using watershed approach and manual quality check step (middle, right). The inference workflow was run on all WSIs to predict NFT subtypes (Pre-NFT or iNFTs), followed by extraction of imaging features used to train and test machine learning models for predicting Braak stages for neuropathology cases. Red circles/boxes represent iNFT annotations, blue circles/boxes represent Pre-NFT annotations. Two Emory cases contained two WSIs for the posterior hippocampus, one from each side of the brain (hemispheres). Overview of project cohorts and specific uses for different cohorts

All WSIs used in this work were stored in an instance of the Digital Slide Archive (DSA) [50]. WSIs were digitized at a resolution of 0.25 microns per pixels (files with SVS extension) or 0.23 microns per pixel (files with NDPI extension), immunohistochemically labeled for tau, and counterstained with hematoxylin. Cases from the Emory University Alzheimer’s Disease Research Center (ADRC) included WSIs from the posterior hippocampus, amygdala, temporal cortex, and occipital cortex. All Emory WSIs were digitized with an Aperio AT2 scanner. Antibodies used to label tau varied, with PHF-1 being the most common (n = 311, kindly provided by Dr. Peter Davies), followed by a pan-tau antibody (n = 73, catalog BYA10741, Accurate Chemical and Scientific, Carle Place, NY), AT8 (n = 51, catalog MN1020, Pierce), and CP13 (n = 12, kindly provided by Dr. Peter Davies). Emory cases were split into two cohorts: Emory-Train (52 cases) and Emory-Holdout (59 cases) (Table 1). Each cohort was used for different parts of the project, as shown in Fig. 1.

**Table 1:** Demographics on neuropathology cohorts used

	Cohorts		
	Emory-Train	Emory-Test	UC Davis
*Demographics*			
Number of cases (M/F)	52 (30/22)	59 (23/36)	23 (12/11)
Average age at death (std. dev.)	70.44 (10.22)	70.83 (14.96)	83.83 (7.32)
*Race/ethnicity*			
Caucasian	42 (80.77%)	48 (81.36%)	18 (78.26%)
Black/African American	10 (19.23%)	7 (11.86%)	1 (4.35%)
Hispanic	–	–	2 (8.70%)
Asian	–	–	1 (4.35%)
Unknown	–	4 (6.78%)	1 (4.35%)
*Braak NFT Stage*			
0	1 (1.92%)	4 (6.78%)	–
I	4 (7.69%)	7 (11.86%)	–
I–II	2 (3.85%)	–	–
II	7 (13.46%)	5 (8.47%)	1 (4.35%)
III	5 (9.62%)	7 (11.86%)	3 (13.04%)
IV	4 (7.69%)	6 (10.17%)	3 (13.04%)
V	7 (13.46%)	4 (6.78%)	5 (21.74%)
VI	22 (42.31%)	26 (44.07%)	11 (47.83%)
*Tau antibody (WSI counts)*			
PHF-1	138 (65.71%)	173 (73.00%)	–
AT8	23 (10.95%)	28 (11.81%)	92 (100%)
CP13	12 (5.71%)	–	–
pan-tau	37 (17.62%)	36 (15.19%)	–

For each cohort we include the demographics (sex, age at death, and race/ethnicity), the number of cases in each Braak NFT stage, and the distribution of WSI stained with different tau antibodies. The NFT Braak stage is provided for each case during neuropathology assessment.

*M* male, *F* female, *std. dev.* standard deviation

A cohort of 23 cases from the University of California-Davis (UC Davis) ADRC was used to test inter-institutional generalizability of Braak NFT stage ML models. Due to differences in neuropathology practice between the institutions, cases from this cohort included the anterior hippocampus in place of the amygdala. Tau staining in this cohort was done using the AT8 antibody (catalog MN1020, Thermo Fisher).

Case inclusion criteria were defined with consultation of a panel of experts (BD, JC, MG) from different institutions. Cases with major infarctions observed during neuropathology assessment in the temporal cortex, occipital cortex, hippocampus, and/or amygdala were excluded.. Cohorts included cases across all Braak NFT stages if available, and with a variety of neuropathology diagnoses, including cases with multiple pathologies present (Table 1 and Additional file 1).

### Braak NFT stage inter-rater agreement analysis

The Emory-Train cohort was used to measure inter-rater agreement for Braak NFT staging in a cohort of five experts with years of neuropathology experience (referred to as raters). All of the experts were individuals who were either board-certified, practicing neuropathologists and/or PhD researchers with greater than 10 years' experience in the area of neurodegenerative disease neuropathology. Raters were recruited from multiple institutions: Emory University (MG, BW), UC Davis (BD), Mt. Sinai University (JC), and Johns Hopkins University (MM). The Braak NFT staging protocol described in Braak et al. [27] was used in this study, and raters were blind to other raters’ analyzes and case demographics. Each rater was provided access to the WSIs through the DSA and used the HistomicsUI viewer to provide Braak NFT stages (Additional file 3: Fig. S1) [50]. HistomicsUI provides capabilities common to most WSI viewers (panning, magnification changes, and rotation), as well as the storage and querying of metadata (e.g. Braak NFT stage), and ability to navigate between WSIs within a collection (e.g. organized by neuropathology case), without the need to store large files locally.

Braak NFT stage inter-rater agreement was measured following the methods described in Montine et al. [28]. Briefly, a weighted Cohen's kappa, using quadratic weights, was used to calculate the agreement between all pairs of raters, reporting the average of all these kappas. Cohen's kappa is used for measuring inter/intra rater agreement of categorical data while taking into account agreement occurring by chance. Strict cut-offs for excellent, good, and poor agreement are not standardized with this approach so we use the general criteria of “excellent” (kappa > 0.75), “good” (0.4 > kappa < 0.75), and “poor” agreement (kappa < 0.4). A bootstrap approach was used to calculate the 95% confidence interval by resampling the cases 1,000 times with replacement. A jackknife approach was implemented to identify outliers in raters, by removing one rater at a time and re-calculating the average weighted Cohen’s kappa [51, 52].

### NFT inter-annotator agreement analysis

**Fig. 2:**
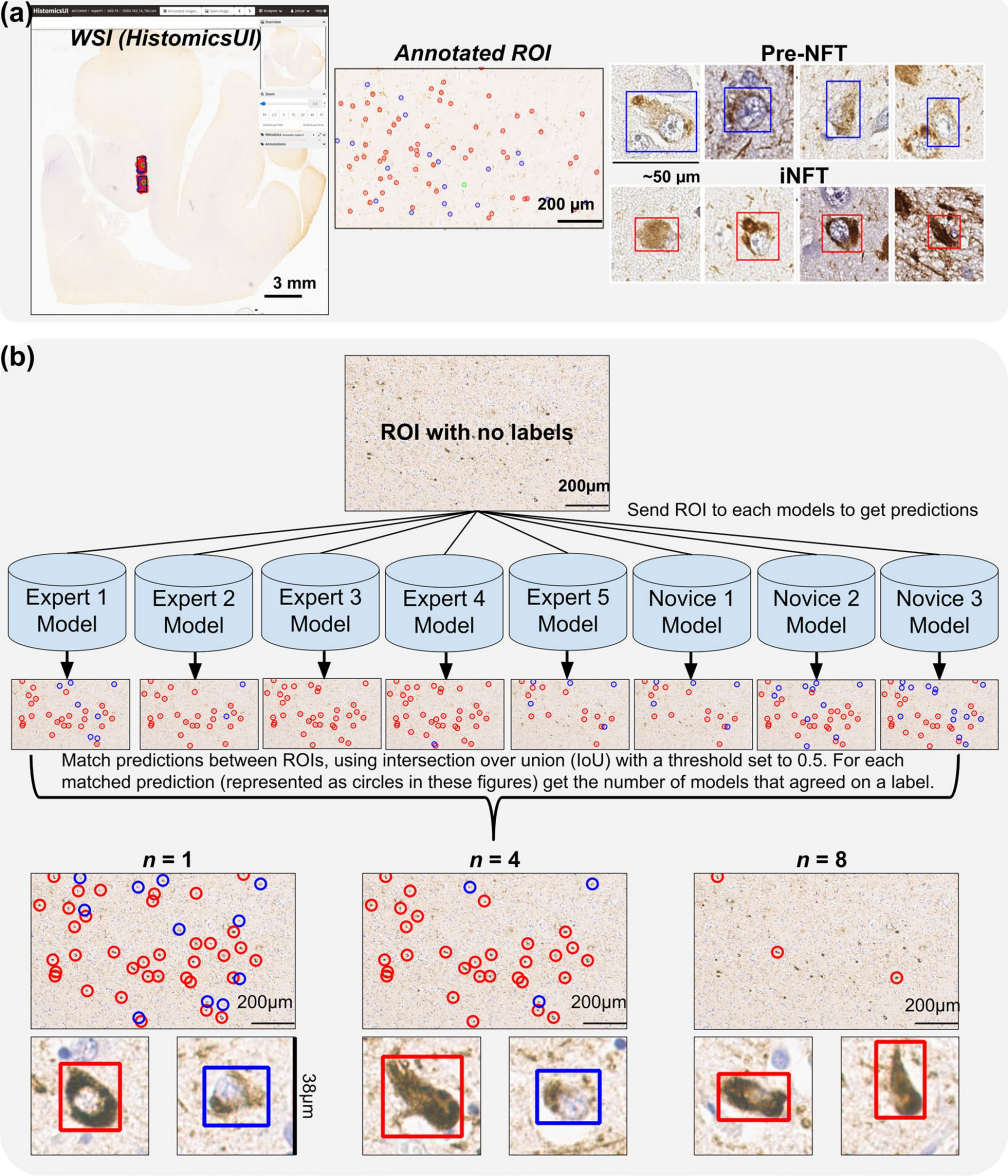
Consensus Labeling of Annotated ROIs. **a** WSI as viewed in the HistomicsUI application, containing annotated ROIs. ROIs are annotated by experts and/or nonvices for Pre-NFTs (blue circles/boxes) and iNFTs (red circles/boxes) using the point annotation tool and these points are converted to bounding boxes using watershed and manual corrections. Green circle in ROI marks a completely annotated ROI. **b** Process used to create labels for unlabeled ROIs using pre-trained models. The best models for each annotator are used as an initial guess of the Pre-NFT/iNFT labels. These sets of predictions are then matched between each other: for each prediction in an ROI, find if any predictions from other ROIs match, using the IoU metric (threshold of 0.5). When creating the final set of labels of the ROI, set a minimum number of models that must agree on a label to be given as the “ground truth”: n. The bottom row of images shows that as n is set higher, the number of labels decreases as more models must agree. Setting n to 1 includes all predictions from all models, with the label (Pre-NFT/iNFT) being set by the label most models agree with. In cases of ties, iNFTs takes precedence over Pre-NFT as the label. Close ups of iNFT and Pre-NFT predictions are also shown with bounding boxes for n = 1, 4, and 8. NFT annotations and workflow for consensus labeling

Additional non-expert/novice annotators were recruited to provide NFT annotations; in total our annotators included the five experts above and four novices. The novices were individuals that at the time of evaluations had  insufficient experience in the area of neurodegenerative disease neuropathology to independently assess a Braak NFT stage. The novice group was made up of an undergraduate student, PhD student (JV), research scientist (EK), and post-doctoral researcher (KF; now an assistant professor). The annotators were tasked with providing WSI annotations for Pre-NFTs and iNFTs in selected regions of interest (ROIs) within the hippocampus and amygdala. The ROIs were selected to include both Pre-NFT and iNFTs from gray matter regions, in relatively high quantities. The criteria used to differentiate Pre-NFTs and iNFTs were developed with consultation from JC and with reference to Augustinack et al. [53]. Instructional material was created and provided to all annotators in the form of a detailed document with image examples and a tutorial video (Additional file 2). Additionally, one-on-one sessions were provided as needed to on-board annotators in the use of the HistomicsUI web application. Participants annotated 15 ROIs from different cases (14 from the posterior hippocampus, one from the amygdala) for all Pre-NFTs and iNFTs present (ROI size varied slightly but was approximately 719 × 1228 µm/3858 × 3853 pixels). HistomicsUI’s point annotation tool was used to allow rapid annotations of the NFTs; the participants were not required to draw boundaries. Participants were blind to each other's annotations (Fig. 2a).

Point annotations were converted to bounding boxes using watershed, and were then manually checked and modified for best fit [54]. Bounding boxes enclosed the tau inclusion and the nucleus when visible. For each pair of annotators a Cohen’s kappa was calculated to measure inter-annotator agreement. The annotations for each annotator were used to create two binary masks for each ROI, a Pre-NFT and an iNFT mask, where 0 are pixels with background and 1 are pixels inside a Pre-NFT or iNFT bounding box. These masks were flattened into a vector and Cohen's kappa was measured for each ROI between pairs of annotators (Additional file 3: Fig. S2). The average Cohen’s kappa of the 15 ROIs was reported for each pair of annotators. We report the results of comparing experts versus experts, experts vs novices, and novices vs novices [55, 56].

### YOLO models trained with human annotated data

To provide ample annotations, an additional 278 ROIs (approximate 719 × 1228 µm/3858 × 3853 pixels in size) were selected from the Emory-Train cohort and randomly assigned to five experts and three of the novices for annotation following the protocol described above (novice four was not included for the rest of the project and only annotated the 15 ROIs needed for NFT inter-annotator analysis). These ROIs, together with the 15 ROIs used in the inter-annotator agreement analysis (total of 293 ROIs), were used to train YOLO (You Only Look Once) models for Pre-NFT & iNFT detection (Additional file 3: Table S1) [57, 58]. We utilized Ultralytics’s YOLOv5 open source implementation as our base and added some project specific modifications [59].

All ROIs were divided into smaller images, which we refer to as tiles, to use in model training and evaluation (1280 × 1280 pixels, with 25% pixel overlap between adjacent tiles, Additional file 3: Fig. S3). The ROIs were grouped by the annotator who labeled them, creating 8 datasets. For each dataset we randomly held-out 10% of WSIs (i.e. all ROIs from these WSIs) for testing. We utilized three-fold cross validation on the remaining data (80% train, 10% validation of WSIs) to avoid performance being dependent on which WSIs are in the train and validation datasets, and report the average of the three folds. The validation datasets were used to prevent overfitting using early stopping (i.e. when performance no longer improved).

An inference workflow was developed to generate predictions on ROIs, which are significantly larger than the tile images. First, the models were used to predict Pre-NFT/iNFT bounding boxes on tiles, and these predictions were merged between overlapping regions (caused by tiling process using 25% overlap) to create final ROI predictions. ROI predictions were compared to the annotator’s ground truth annotations using the intersection over union (IoU) at a threshold of 0.5 to calculate micro-F1, macro-F1, precision, and recall metrics. All models were trained using multi-GPU data processing with a batch size of 24 and two GPUs (NVIDIA A4500s or A5000s). All models were trained to 100 epochs with early stopping after 20 epochs of no improvement on the validation dataset.

### Emory-Holdout dataset ROIs

A dataset of 28 annotated ROIs was selected from the Emory-Holdout cohort and used as the consensus test dataset for YOLO ML models. The ROIs were chosen specifically to include all brain regions and Braak NFT stages equally (4 brain regions and stages 0 to VI). These ROIs were annotated by JV and checked for correctness by an expert (TP) (Additional file 3: Table S2).

### Consensus labeled datasets and models

The best models for each annotator (n = 8) were leveraged to predict tentative labels for an additional set of large ROIs (n = 194, ~ 1438 × 2256 µm/5752 × 9024 pixels) from the Emory-Train cohort. These ROIs were taken from WSIs with previous ROIs but on different parts of the image, as well as WSIs without any previously annotated ROIs. For each ROI a set of tentative labels was predicted, one from each model, and a consensus strategy was implemented to finalize the labels (Fig. 2b). We tested the performance of training models with labels created from an *n*-consensus model agreement. For example: for a given ROI we predicted labels for the best annotator models, based on performance on the Emory-Holdout dataset. These labels were then combined into a single set of predictions by a *n*-agreement of models. To do this we calculated the intersection-over-union (IoU) between prediction boxes of different models and identified boxes that overlapped sufficiently (IoU threshold of 0.5). For a given set of overlapping boxes we calculated the most frequently occurring label: Pre-NFT or iNFT. If the label’s consensus count was above *n*, then that box is assigned the consensus label as the ground truth. We tested this for *n* of 1 (take predictions from all models) to 8 (all models must agree). In cases of ties, an iNFT label took precedence over a Pre-NFT label.

This workflow was also repeated on ROIs previously annotated to create a dataset totaling 487 ROIs, labeled in the same manner. We used three-fold cross-validation (90% of WSIs for train, 10% for validation) to train a new set of YOLOv5 models. These new models were evaluated on the Emory-Holdout dataset for performance comparison to models trained on data labeled by single annotators.

### Model assisted labeling

To improve model performance while minimizing annotation time, we implemented a model-assisted-labeling workflow that incorporates Python code, the HistomcisUI viewer, and a custom Javascript application that integrates with the DSA [50]. We started with labels created by *n* of 4 consensus models, as described above. The models predict the bounding box of the object, the label (Pre-NFT or iNFT), and a confidence score. For each ROI we calculated the average confidence of the predictions, by averaging the confidence of all predictions of that ROI. We then iterate by selecting 25 of the ROIs with the lowest average confidence (approximately 5% of total ROIs), and manually reviewing and revising the labels as needed. To do this we pushed the boxes as annotations to HistomicsUI, and used a combination of HistomicsUI and a custom-developed web application to (1) adjust box boundaries, (2) change label between Pre-NFT and iNFT, (3) delete false predictions, and (4) add missed Pre-NFTs/iNFTs. We then updated the labels before training a new iteration of models. These new models were then used to update the labels on ROIs that have not been curated in previous iterations. The next set of 25 ROIs are selected in a similar fashion and the process is repeated. We repeated this process until performance on the Emory-Holdout dataset was shown not to improve. Additional models were also trained using only the ROIs that were manually curated after the final iteration as well as models trained to specifically predict NFTs in each region (Additional file 3: Fig. S4).

### WSI inference and background ROIs

The inference workflow described above was extended to work on entire WSIs. Briefly, we split an entire WSI into small tile images of 1280 × 1280 pixels with a stride of 960 pixels (25% overlap between adjacent tiles) and saved the images locally. To speed the workflow we only processed tile images containing tissue, by referencing a tissue mask pre-calculated using HistomicsTK’s tissue detection workflow (https://github.com/DigitalSlideArchive/HistomicsTK). A pre-trained model was then used to predict Pre-NFTs/iNFTs in all saved tiles. The predictions were saved as coordinates and merged together using a combination of non-max-suppression with IoU threshold of 0.45 and a custom approach to remove boxes mostly contained in other boxes (IoU threshold of 0.7). This approach was necessary to remove duplicate predictions caused by overlapping images and remove small predictions contained in larger ones. The final prediction boxes were then pushed as DSA annotations for review and modification. Using HistomicsUI we then identified regions of tissue with high rates of false positive errors, and added additional ROIs from these regions manually selected to contain no Pre-NFT/iNFTs in order to enhance the training data. We trained new models with these additional ROIs to reduce the number of false positives.

### ML Braak NFT staging

The best model-assisted-labeling model was chosen based on top performance on the Emory-Holdout dataset and was used to predict Pre-NFTs and iNFTs on all WSIs (Emory-Train, Emory-Holdout, and UC Davis cohorts). Quantitative histologic features were then extracted for each WSI which included the density of Pre-NFTs/iNFTs in the tissue (number of prediction boxes normalized by tissue area) and the highest number of Pre-NFT/iNFTs in a field of view (FOV) of 4 mm^2^. The FOV was chosen to mimic the area observed when using a 10× lens at a microscope with tissue slide, the common approach neuropathology practice.

Average clustering coefficient is a graph theory concept measuring how close objects are to each other in a population of nodes (Pre-NFT/iNFT prediction in our case). To evaluate items in this manner, we first define a maximum radius that two objects must be from each other to be considered “connected.” A previous study reported the average clustering coefficient of iNFTs accurately predicted cognitive impairment when the radius was between 150 and 600 µm [38]. Following this we calculated the average clustering coefficient for Pre-NFT and iNFTs on the selected FOV at various radii (150 to 600 microns in 50 micron intervals) and added it to our feature list.

For each case we used a feature list of size 88 and used recursive feature elimination to narrow down the features to the 20 most critical for the task of Braak NFT staging (Additional file 3: Table S3). A random forest classifier (scikit-learn Python package) was trained on this subset of features to predict Braak NFT stages [60]. We used a random grid search approach to tune the hyper-parameters for our dataset and we report performance as the weighted Cohen’s kappa (quadratic distance). The Emory-Holdout cohort was used to train the models and the Emory-Train cohort, as well as the UC Davis cohort, to test predictions. We did this for two reasons: (1) the Emory-Train cohort had only one case of stage 0 and (2) this allowed us to compare performance against Braak NFT stages provided by expert raters. Predicted Braak NFT stages were also compared against the Braak NFT stage provided in the original autopsy report.

Additionally, we compared the density of Pre-NFT/iNFT by region and stage to identify patterns and statistical significance between groups. Statistical analysis was done using Python’s statsmodel package, implementing a one-way ANOVA between groups with post-hoc Tukey’s test for comparison between cohorts, using a significance value of 0.05 for all tests [61].

## Results

### Braak NFT stage inter-rater agreement

**Fig. 3:**
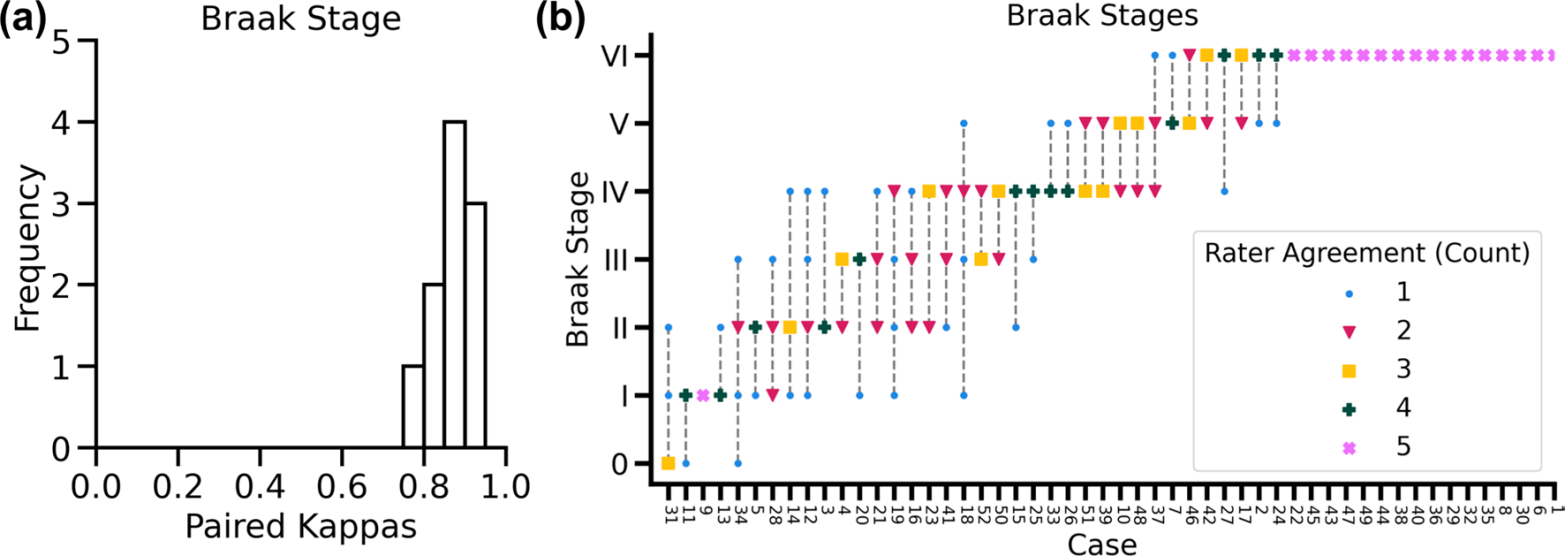
Braak stage inter-rater agreement on the Emory-Train cohort (52 cases). **a** Agreement between pairs of annotators was excellent, left histogram. **b** The right figure shows Braak stages provided for each case by 5 expert raters, sorted by the most common stage given for each case for better readability. The number of raters providing a Braak stage is represented by a different marker/color (see legend). Vertical dotted lines are added for readability. Braak stage inter-rater agreement

The Emory-Train cohort, consisting of 52 cases from Emory University, was used to measure Braak NFT stage inter-rater agreement between five experts in neuropathology (Table 1). Each case included a tau-stained WSI from the posterior hippocampus, amygdala, temporal cortex, and occipital cortex regions (210 WSIs in total, see Fig. 1). We measured inter-rater agreement using the weighted Cohen’s kappa with quadratic weights, penalizing disagreement more when farther apart from each other. Braak NFT stage inter-rater agreement showed a weighted Cohen’s kappa of 0.88 (95% CI 0.82–0.91) across these raters. Cases with higher Braak NFT stages showed better agreement among raters compared to low to intermediate Braak NFT stages. Perfect agreement was observed in 17 of 52 cases, with 16 of these cases being rated the highest stage (Fig. 3).

### NFT inter-annotator agreement analysis

**Fig. 4:**
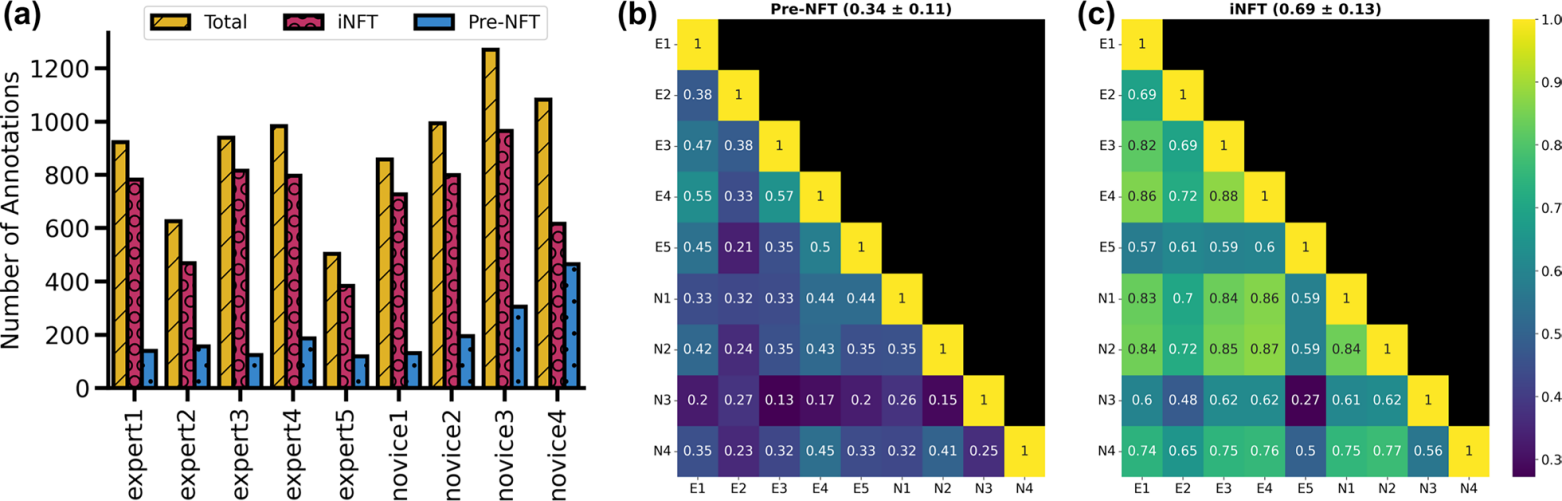
Pre-NFT/iNFT inter-annotator agreement analysis. **a** Count of annotations in the 15 ROIs of hippocampal and amygdala areas for each annotator. Heatmaps of pair Cohen’s kappa between annotators for Pre-NFT (**b**) and iNFT (**c**). The title shows the average Cohen’s kappa for the heatmap with the standard deviation. N: novice, E: expert. Inter-annotator Agreement Analysis for Pre-NFT/iNFT Detection

Inter-annotator agreement for the task of NFT detection was measured on a set of 15 rectangular regions of interest (ROIs) in WSIs (Fig. 2a). ROIs were annotated by five experts and four novices for Pre-NFTs and iNFTs. The mean Cohen’s kappa varied greatly between annotators and showed better agreement for iNFTs (0.69 ± 0.13) than Pre-NFTs (0.34 ± 0.11) (Fig. 4b, c). Outliers were seen in both experts and novices (Fig. 4a).

The mean Cohen’s kappa was computed between specific groups of annotators, comparing experts against other experts, novice against other novices, and experts against novices. Pre-NFT agreement between experts was the highest at a Cohen’s kappa of 0.42 ± 0.11, compared to expert vs. novices (0.31 ± 0.11) and novices vs. novices (0.29 ± 0.11). Agreement for iNFTs, however, was similar between all comparison groups: expert versus expert (0.70 ± 0.11), expert versus novice (0.68 ± 0.15), and novice vs novice (0.69 ± 0.10).

### YOLO models trained with human annotated data

A cohort of five expert and three novice annotators (novice four excluded) looked at approximately 50 ROIs from multiple cases, with only 15 ROIs being shared between all annotators for inter-annotator agreement, described above (Additional file 3: Table S1 & S4). These sets of ROIs were selected from the Emory-Train cohort and used to train YOLO object detection models for each annotator. Performance was measured by calculating the number of true positives, false positives, and false negatives on the ROIs after inference of the models. We reported F1-scores by class and account for class imbalance with the macro-F1 score.

Model performance was better at predicting iNFTs than Pre-NFTs (Table 2). Validation and Test dataset performance was similar for most models, and this metric reflects how well the models learned the prediction patterns of specific annotators. An additional dataset of 28 ROIs, from a secondary Emory-Holdout cohort, was labeled by two annotators as a consensus test dataset (Table 1 and Additional file 3: Table S2). Performance on the Emory-Holdout dataset shows how good each annotator model was at predicting on a consensus labeled dataset. Pre-NFT performance was poor for all models, with the top performing models being from expert 3 at an F1-score of 0.31 ± 0.08. iNFT performance was medium to poor, with the highest performing models being expert 3 at an F1-score of 0.66 ± 0.03. All model performance was reported as the average of three-fold cross validation, with standard deviations (Table 2).

**Table 2:** Results for YOLO models trained with data annotated by humans

Annotators	Pre-NFT F1 Score			iNFT F1 Score			Macro F1 Score		
	Val	Test	Emory Holdout	Val	Test	Emory Holdout	Val	Test	Emory Holdout
Novice 1	0.49 ± 0.12	0.63 ± 0.10	0.20 ± 0.09	0.76 ± 0.02	0.76 ± 0.02	0.59 ± 0.05	0.63 ± 0.07	0.70 ± 0.06	0.39 ± 0.02
Novice 2	0.44 ± 0.04	0.39 ± 0.06	0.21 ± 0.08	0.80 ± 0.01	0.73 ± 0.02	0.51 ± 0.05	0.62 ± 0.02	0.56 ± 0.02	0.36 ± 0.06
Novice 3	0.67 ± 0.08	0.65 ± 0.03	0.13 ± 0.02	0.65 ± 0.05	0.74 ± 0.01	0.59 ± 0.01	0.66 ± 0.06	0.70 ± 0.02	0.36 ± 0.01
Expert 1	0.36 ± 0.08	0.55 ± 0.05	0.21 ± 0.04	0.75 ± 0.06	0.75 ± 0.00	0.45 ± 0.06	0.55 ± 0.06	0.65 ± 0.03	0.33 ± 0.05
Expert 2	0.41 ± 0.24	0.26 ± 0.13	0.10 ± 0.01	0.64 ± 0.11	0.46 ± 0.03	0.39 ± 0.10	0.53 ± 0.09	0.36 ± 0.07	0.25 ± 0.05
Expert 3	0.40 ± 0.24	0.54 ± 0.04	0.31 ± 0.08	0.80 ± 0.05	0.75 ± 0.03	0.66 ± 0.03	0.60 ± 0.11	0.65 ± 0.03	0.48 ± 0.05
Expert 4	0.49 ± 0.21	0.40 ± 0.02	0.29 ± 0.07	0.79 ± 0.01	0.71 ± 0.02	0.59 ± 0.07	0.64 ± 0.10	0.55 ± 0.01	0.44 ± 0.06
Expert 5	0.47 ± 0.02	0.43 ± 0.06	0.17 ± 0.04	0.67 ± 0.04	0.65 ± 0.01	0.38 ± 0.04	0.57 ± 0.03	0.54 ± 0.03	0.28 ± 0.03

The Emory-Holdout 28 ROI dataset is the consensus annotated dataset from a hold-out Emory cohort. Val (Validation) and Test datasets are annotated by the specific annotator and reflect how well the models learned the annotator nuances. All values reported are the average results of three-fold cross-validation models for each annotator. Standard deviations are shown

### Models trained on datasets labeled by *n-*consensus

An additional set of large unlabeled ROIs (~ 1438 × 2256 µm, 5752 × 9024 pixels) from the Emory-Train cohort were added to the original dataset of ROIs. For each set of annotator models (models trained on datasets annotated by a single annotator) we chose the model with the best performance on the Emory-Holdout dataset and predicted labels on these ROIs. For each ROI we combined the various model predictions using a majority voting scheme with an agreement requirement, where *n* is the number of models that must agree to add a consensus label (Fig. 2b). We trained these models and evaluated the performance on the Emory-Holdout dataset (Additional file 3: Fig. S5). Best model performance was seen when *n* was set to two (0.59 macro F1 score), and performance decreased as the value of *n* increased thereafter.

### Model-Assisted-labeling

We utilized a model-assisted-labeling workflow to improve the data quality and consequently demonstrate an increase in overall model performance. Using custom Python code, the HistomicsUI annotation viewer, and a custom web application, we iteratively improved and refined the labels of the training dataset (Additional file 3: Fig. S4). We used the best performing consensus labeled model, when *n* was set to four, and predicted labels on all our training ROIs. We then looked at the ROIs with the lowest prediction confidence, and curated the labels. The HistomicsUI viewer and custom web application allowed us to view predictions directly on WSIs as annotations, and quickly apply changes to the labels. Label curation involved adjusting bounding boxes on predictions, deleting false positives, adding missed Pre-NFTs/iNFTs, and re-labeling misclassified Pre-NFTs/iNFTs. We then froze these labels so they would not be modified again, and trained a new model. This new model was used to predict a new set of labels and the next set of ROIs were then curated, with each iteration curating 5% of total ROIs.

Model performance using model-assisted-labeling initially improved, but plateaued after the fourth iteration (20% of ROIs curated). Performance improvement was mainly due to increased accuracy in predicting iNFTs (from 0.67 to 0.77 F1-score), while Pre-NFT performance remained mostly unchanged (0.38 F1-score). After the eighth iteration (40% of ROIs curated) we trained a model using just the curated ROIs (n = 200) and saw improvements to the performance on the Emory-Holdout dataset (top performance was 0.62 macro F1-score) (Table 3). Confusion matrices revealed that these improvements were mostly due to a lower number of false negatives, compared to the base consensus models (Additional file 3: Fig. S6).

**Table 3:** Model performance on the Emory holdout dataset for model-assisted-labeling models

Models	Pre-NFT			iNFT			Macro F1-score
	Precision	Recall	F1 score	Precision	Recall	F1 Score	
iter. 1	0.36 ± 0.03	0.40 ± 0.03	0.38 ± 0.03	0.86 ± 0.01	0.57 ± 0.00	0.69 ± 0.00	0.53 ± 0.01
iter. 2	0.37 ± 0.02	0.45 ± 0.01	0.41 ± 0.01	0.84 ± 0.02	0.63 ± 0.01	0.72 ± 0.01	0.56 ± 0.01
iter. 3	0.29 ± 0.02	0.46 ± 0.01	0.36 ± 0.02	0.82 ± 0.01	0.71 ± 0.02	0.76 ± 0.01	0.56 ± 0.01
iter. 4	0.31 ± 0.02	0.47 ± 0.01	0.37 ± 0.02	0.79 ± 0.01	0.74 ± 0.02	0.77 ± 0.01	0.57 ± 0.00
iter. 5	0.31 ± 0.03	0.51 ± 0.00	0.38 ± 0.02	0.78 ± 0.01	0.76 ± 0.02	0.77 ± 0.01	0.58 ± 0.02
iter. 6	0.30 ± 0.04	0.53 ± 0.02	0.38 ± 0.03	0.75 ± 0.01	0.78 ± 0.02	0.77 ± 0.01	0.57 ± 0.02
iter. 7	0.29 ± 0.01	0.53 ± 0.02	0.38 ± 0.02	0.74 ± 0.00	0.81 ± 0.01	0.77 ± 0.00	0.58 ± 0.01
iter. 8	0.26 ± 0.01	0.54 ± 0.04	0.35 ± 0.02	0.73 ± 0.02	0.80 ± 0.02	0.76 ± 0.02	0.56 ± 0.02
amygdala	0.46 ± 0.06	0.52 ± 0.08	0.48 ± 0.00	0.73 ± 0.03	0.86 ± 0.02	0.79 ± 0.03	0.64 ± 0.02
hippocampus	0.27 ± 0.04	0.44 ± 0.08	0.33 ± 0.04	0.68 ± 0.03	0.78 ± 0.01	0.73 ± 0.02	0.53 ± 0.03
temporal	0.14 ± 0.06	0.20 ± 0.10	0.16 ± 0.07	0.76 ± 0.06	0.67 ± 0.05	0.71 ± 0.04	0.44 ± 0.06
occipital	0.04 ± 0.03	0.22 ± 0.19	0.06 ± 0.05	0.68 ± 0.09	0.76 ± 0.09	0.71 ± 0.04	0.39 ± 0.05
QC ROIs	0.41 ± 0.04	0.45 ± 0.01	0.43 ± 0.03	0.78 ± 0.01	0.85 ± 0.03	0.81 ± 0.01	**0.62 ± 0.02**
best consensus	0.36 ± 0.04	0.53 ± 0.03	0.43 ± 0.04	0.82 ± 0.01	0.70 ± 0.01	0.76 ± 0.01	0.59 ± 0.02

Additional models are also shown which are modifications to the datasets used. iter.: iteration in model-assisted-labeling, amygdala/hippocampus/temporal/occipital: models trained on ROIs only from specific regions of the brain (temporal and occipital refers to the temporal and occipital cortex), QC ROIs: models trained only with ROIs with curated labels during model-assisted-labeling, best consensus: consensus model when *n* equal to 4 (Additional file 3: Fig. S4). Values are shown with standard deviation from the average of the three-fold cross-validation models. Bold score is the best performing model trained on the dataset from all brain regions

Models were also evaluated by brain region, training only on curated ROIs from specific brain regions and evaluating on the subset of the Emory-Holdout dataset from these regions. Models trained on ROIs from amygdala demonstrated the highest performance with a macro F1-score of 0.64 ± 0.02, while the worst performing region model was the occipital cortex with a macro F1-score of 0.39 ± 0.05 (Table 3).

### WSI inference

**Fig. 5:**
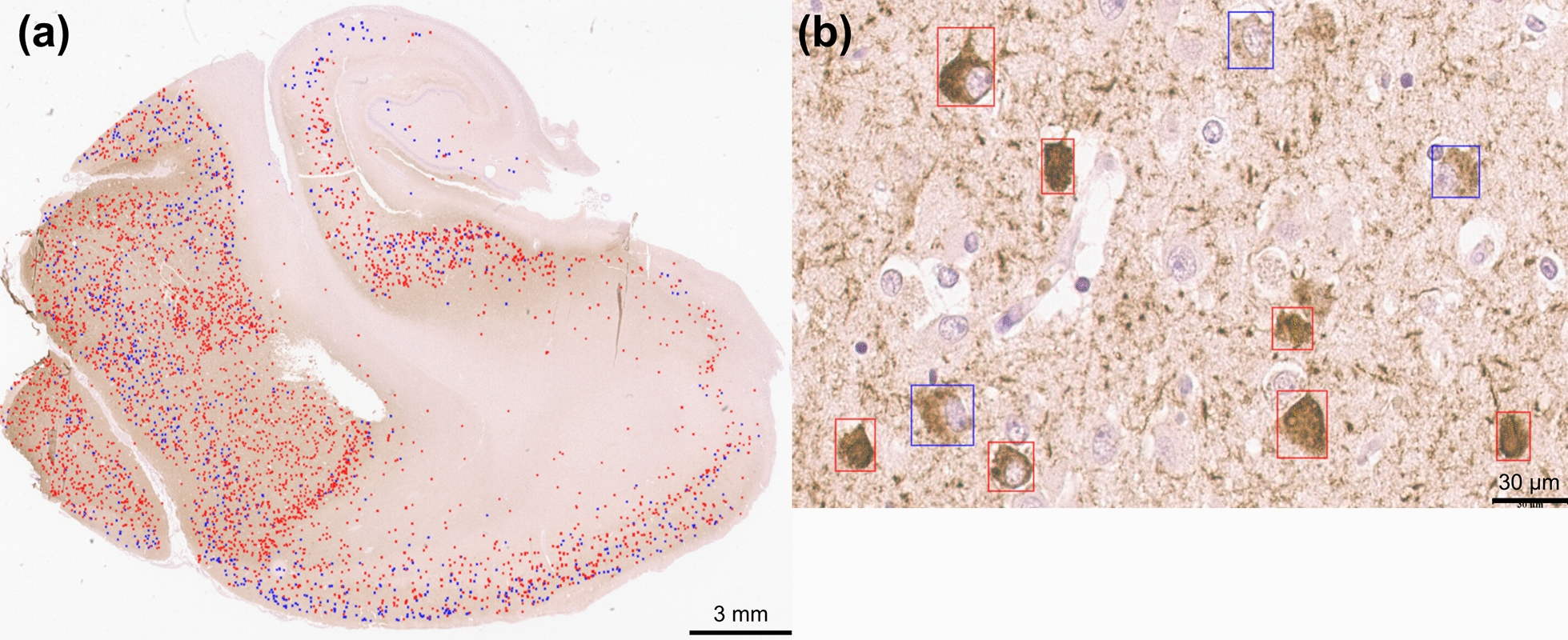
Inference results for NFT detection. **a** Example WSI from the Emory-Holdout cohort with Pre-NFT (blue) and iNFT (red) predictions. Predictions are mostly localized to the gray matter regions of the tissue (outer edge), as is expected for NFTs since neurons are mostly present in these regions. **b** At high resolution we can see the distinct differences between the two classes of predictions, with iNFTs being more fibrillary and darker in color while Pre-NFTs being putative in texture, showing a clear nuclei, and a lighter brown. NFT Detection on WSIs

The best trained model, as determined by performance on the Emory-Holdout dataset, was used to predict Pre-NFTs/iNFTs on entire WSIs. Pre-NFT/iNFT predictions were mostly confined to gray matter regions of tissue, where most neurons are found (Fig. 5). Inference time varied greatly between WSIs based on the number of objects detected, with the most time consuming step being predicting the labels. The fastest time to inference was seven minutes and predicted a total of 1266 Pre-NFTs/iNFTs, while the slowest WSI inference time was 46 min and predicted a total of 32,867 Pre-NFTs/iNFTs (Additional file 3: Fig. S7). Inference time on the full 52 case Emory-Train cohort (total 210 WSIs) was 67.9 h using a single server and two NVIDIA RTX A4500 GPUs.

**Fig. 6:**
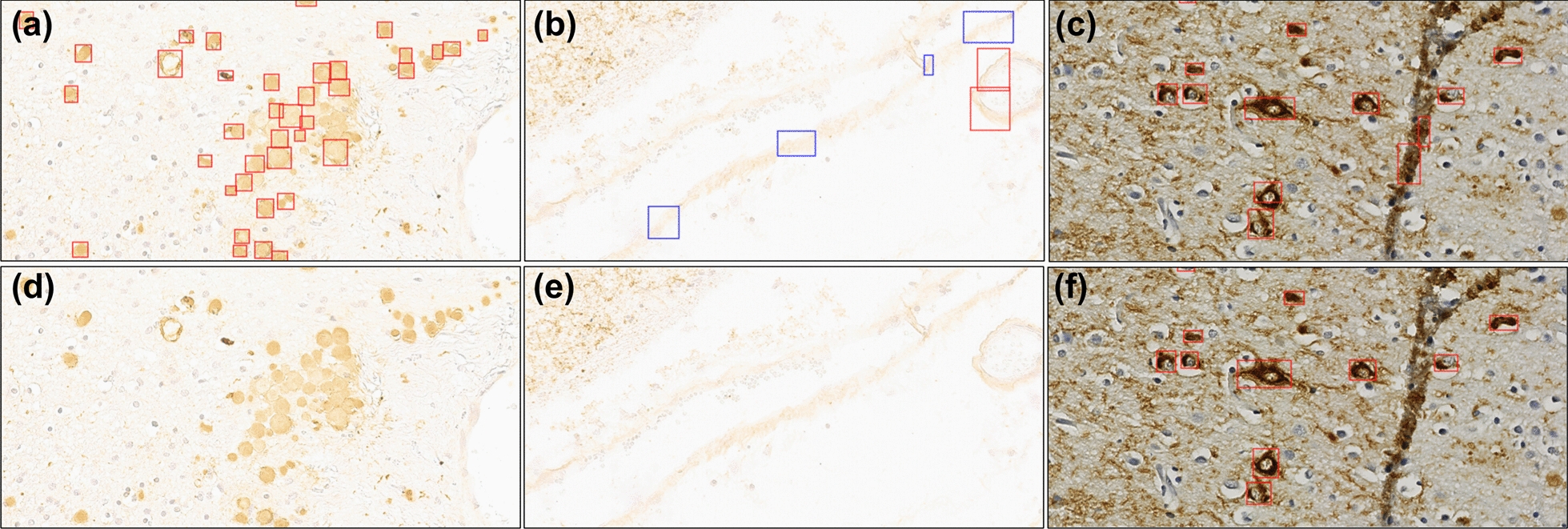
Inference results before (top images **a**, **b**, and **c**) and after (bottom images **d**, **e**, and **f**) training with background ROIs. Examples are shown of the models learning new features and what to ignore as background objects that are not Pre-NFTs/iNFTs. **a** and **d** Models learn to ignore corpora amylacea. In the training dataset there were no examples of these objects and thus were originally predicted as NFTs. **b** and **e** Edges or vessels are also often predicted as Pre-NFTs or iNFTs since the model was never exposed to these during training, but can learn to ignore these as background. **c** and **f** Folded tissue was also a common mistake as it provided a sudden darker shade compared to background, oftentimes having edges that look of NFT shape. However, new models learn to ignore these after seeing examples of folded tissue. Red boxes: iNFTs, blue boxes: Pre-NFTs. Inference Mistake and Corrections for WSIs

Inference results were pushed to HistomicsUI for visualization at the WSI level (Fig. 5a). To aid in quality control, we examined results across our training dataset to identify errors the models appeared to be making consistently. Objects of similar morphology, edges of tissue, and tissue artifacts, such as folds, consistently led to false positive predictions (Fig. 6a–c). Using HistomicsUI we added a new set of ROIs to the training dataset containing examples of these false positive predictions but no Pre-NFTs/iNFTs. New models trained on this expanded training dataset maintained similar performance on the Emory hold-out dataset (28 ROIs), and new inference results showed a significant decrease in false positives around edges of tissue and folds. Additionally, similar, but task irrelevant pathology found in some WSIs was more consistently ignored (Fig. 6d–f).

### Predicting Braak NFT stages with imaging features

**Fig. 7:**
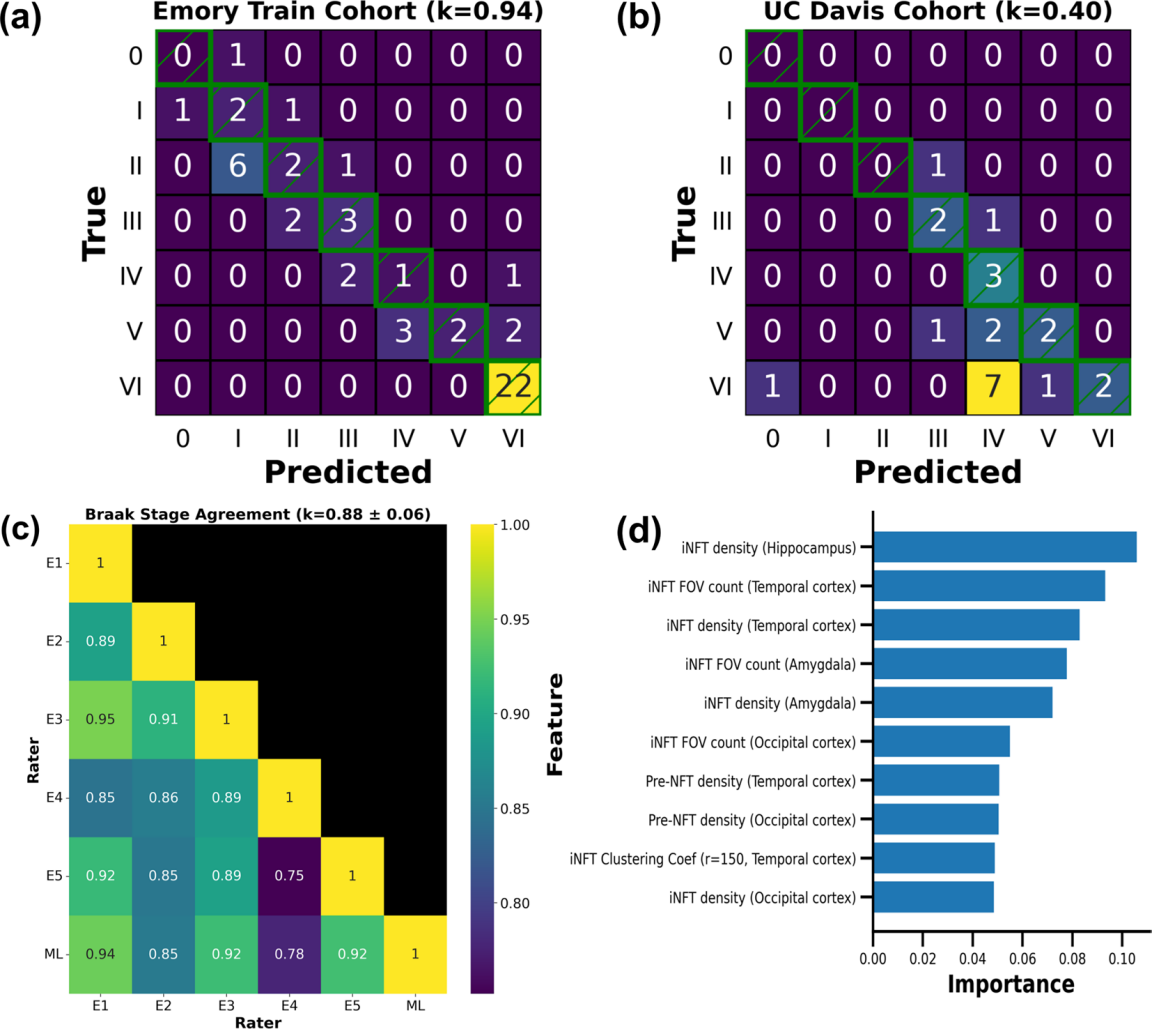
Braak NFT stage prediction results using imaging features. **a** Prediction results on the Emory-Train cohort when compared against the Braak NFT stage assigned during initial neuropathology autopsy, 52 cases. **b** Prediction results on the UC Davis cohort, 23 cases. Green boxes with hatches are used to highlight the diagonal. The weighted Cohen’s kappa is shown in the title. **c** Heatmap of weighted Cohen’s kappa for the Emory-Train cohort between pairs of expert raters and the ML model. The average and standard deviation of all Cohen’s kappas is shown in the title. **d** Top 10 most important features for predicting Braak stages. The random forest classifier reports the feature importance, with the feature value (x-axis) being a normalized value where the sum of all feature importances equals 1. E: expert, ML: random forest ML classifier, k: weighted Cohen’s kappa, r: radius used when calculating the average clustering coefficient, FOV: field of view (see methods, ML Braak NFT Staging section), coef: coefficient. Predicting Braak NFT Stages with Imaging Features and ML

Random forest classifiers (scikit-learn Python package) were trained to predict Braak NFT stages using a set of imaging features created from WSI inference results [60]. While experts typically use details such as anatomical region when assessing Braak NFT stage, no additional annotations were added when creating these imaging features. To allow comparison against Braak NFT stages from our five experts, we reversed the cohorts used in training and testing. The model was trained on the Emory-Holdout cohort of 59 cases and evaluated on the Emory-Train cohort, as well as a separate cohort from UC Davis (23 cases) (Table 1). Performance was reported as a *weighted* Cohen’s kappa, similar to those reported in the inter-rater agreement analysis above. Agreement on the Emory-Train cohort was comparable to those seen in neuropathology agreement analysis (Fig. 7a–c).

In contrast, agreement on the UC Davis cohort was comparatively low, with poor overall performance (Fig. 7b). We inspected the WSI inference in the cohorts by plotting the average counts of iNFT and Pre-NFT predictions on cases with varying amounts of tau pathology (grouped by the Braak NFT stage provided during neuropathology diagnosis). There are more iNFTs than Pre-NFTs predicted in a WSI from the Emory cohort on average, with the opposite being true of the UC Davis cohort. We also observed a smaller number of predictions on the UC Davis cohort overall (Additional file 3: Fig. S8).

## Discussion

One of the biggest challenges for ML in highly technical domains, such as medicine, is the lack of large diverse well annotated datasets. Our approach allowed us to circumvent this by minimizing the human annotations required, through use of unique “individual” models to label a large number of images. We demonstrate a workflow which generates large datasets of well-annotated neuropathology images, by effectively augmenting annotations generated by domain experts. However, we also show, even with detailed instructions, annotations from different experts can vary considerably. We effectively counter this general variability using ML models trained to mimic the unique nuances and opinions of the annotators. These models are capable of creating a much larger, yet robust, training dataset which circumvents the inter-annotator variance by incorporating a consensus decision process. We show with this approach, even novice annotators can provide valuable data, depending on the complexity of the task. By utilizing a model-assisted-labeling workflow to iteratively improve labels in an interactive manner, we further demonstrate the relative ease with which accuracy of the dataset may be improved, while also reducing overall burden on the expert (Additional file 3: Fig. S9).

While previous works share similarities with this study, they do not address the issue of inter-rater annotator disagreement, nor do they demonstrate implementation of a workflow which puts these approaches into practice for neuropathology use. Current approaches often take several hours to run on a single WSI and are not easily viewed in a setting that is familiar or comfortable for experts (i.e. neuropathologist/neuropathology researchers), and it is often unclear how those workflows could be improved without considerable re-engineering of the original approach.

The results of the inter-annotator agreement analysis for NFT detection herein showed significant disagreement between both novice and expert neuropathology annotators. We specifically attempted to remove personal experience and institutional biases from the task of NFT annotations by providing clear guidelines on what defines as a Pre-NFT/iNFT in terms of this study. However, even with these precautions the agreement was poor, showing very different quantities of annotations, and often very different annotations in general, provided on the same 15 ROIs (Fig. 4).

We included two classes of tau inclusions in this analysis that span different stages of NFT progression. The Pre-NFT stage occurs before the formation of mature intraneuronal NFTs, and it is still not clear the importance of this early stage and if it could be leveraged therapeutically, while the iNFT stage is the traditional mature phase of NFT formation, with a clear inclusion with fibrillary texture that often crowds the nucleus and pushes to the edge of the soma [53, 62]. Extracellular or ghost tangles were not included in this study. The two most common antibodies used in this project, PHF-1 and AT8, do not readily stain for these tangles in Emory and UC Davis cohorts, although some studies have noted them to stain extracellular tangles [8, 53]. There may also be protocol differences that may alter staining (e.g., formic acid pre-treatments) and/or differences in cohort composition as there was a dearth of lower Braak NFT stages in the UC Davis cohort. Alternative approaches, such as histological staining with hematoxylin and eosin which can aid in visualization of ghost/extracellular tangles could be used in future studies to include this third stage. In theory, NFT stages are clearly different from each other, however the reality is the process of NFT progression is dynamic and there exist stages that incorporate aspects of both Pre-NFT and iNFTs, as well as the later “ghost tangle” phase (not evaluated in the current study). This creates instances of ambiguity as to what stage an inclusion should be classified under. The tau antibody used for staining also plays an outsized role in determining which stage of NFT formation is most predominantly stained, and therefore the stage which is emphasized in a given slide or section of tissue, which, for obvious reasons, has a strong influence on the later results of this study [8]. Specifically, the Emory cohorts were typically stained using an antibody which binds to the PHF-1 protein and preferentially stains iNFTs over Pre-NFTs [8]. This was clearly reflected in the counts we obtained throughout the project, with iNFTs being considerably more abundant than Pre-NFTs in this cohort (Fig. 4a and Additional file 3: Table S1). Agreement analysis also reflected this, with agreement for Pre-NFTs roughly half of that seen for iNFTs (Fig. 4b).

We acknowledge the agreement analysis for Pre-NFT/iNFT detection can likely be improved by providing a more detailed set of instructions to annotators (Additional file 2). Efforts were made to uncover the root cause(s) of the differences observed, and upon follow up discussions with annotators, it was discovered these deviations were likely at least partially caused by a misunderstanding(s) of the instructional material. While this may not have been an issue with the experts, it happened on two occasions for novices. Novice 3, who had no experience in this field, simply labeled any brown morphology/object as an iNFT, even when these proposed iNFTs were not large enough to be consistent with the given criteria, and were demonstrably similar to background staining present elsewhere in the tissue. Similarly, novice 4 misinterpreted one aspect of the instructions that specified vacuoles *might* be visible in Pre-NFTs, as an explicit criteria (i.e. need to be visible to class as Pre-NFT). As a result their annotations showed comparatively higher numbers of Pre-NFTs, whose putative texture can be misinterpreted as vacuoles in regions of high background staining.

Our primary reason for including an inter-annotator agreement analysis was to quantify if novice annotations could match experts, given limited instruction. In contrast to the above, novices 1 & 2 showed comparable agreement with several of the experts. Two expert outliers were also identified, experts 2 & 5 were both more stringent in their definition of iNFTs (Fig. 4a). This was not due to a misunderstanding of the instructional materials, as occurred for novices, but instead with personal experience and preferred nuances in their definitions of NFTs.

It was important for us to determine, through quantification, if the ML workflow we developed would successfully learn the nuances of the annotators with high fidelity. The first implementation of this workflow showed models trained in this framework could learn the subtleties of individual annotators, but were not able to perfectly recreate their decision making process (Table 2). Specifically, as was consistent throughout this study, Pre-NFT detection was relatively poor. We hypothesize this is largely due to the lower number of Pre-NFTs in the training datasets compared to iNFTs, as well as the antibody used to stain the majority of the WSIs used for this study, as it does not preferentially, or even equivalently, stain for this NFT stage. Another challenge is the considerable background staining and abundance of neuropil threads found in our dataset (Additional file 3: Fig. S11). On WSIs with heavy, non-specific staining, it becomes difficult to differentiate an inclusion as being Pre-NFT or iNFT. However, these WSIs were included intentionally in order to more accurately represent the variability of tissue slides common in neuropathology practice, and to allow us to gauge the utility of, and identify challenges when developing the workflow.

Performance on the Emory-Holdout dataset, composed of 28 ROIs and including a balanced number of Braak NFT stages (including Braak stage 0) and brain regions (Additional file 3: Table S2 and Fig. S12), was poor for models trained using human annotated datasets. This test dataset was initially annotated by JV (novice) and was subsequently evaluated for correctness, based on the criteria set forth in this study, by TP (expert with familiarity with the study), who also added any relevant modifications to improve quality. It is difficult, and maybe impossible at this point, to say if a single expert or groups of experts can provide a “true” label for NFTs. However, we considered this an effective approximation and used this dataset as a target to aim for when implementing our ML workflows. The poor performance of the initial models on this dataset was not surprising, as the models where trained on the nuances of specific annotators and not the consensus labels.

ML requires large datasets, especially when the task is difficult, complex, contains much subtlety/nuance, or would otherwise rely on some feature of the human brain for which we do not yet have a clear computational equivalent. A task like computational NFT detection can be easy, when the datasets being used for training and inference appear very homogeneous. However, neuropathology datasets are often very heterogeneous with significant variations in staining, tissue morphology, pathologies present, and potential tissue artifacts included.

To address this in part, we have herein demonstrated a model-assisted-labeling workflow that can be used to leverage our pre-trained, annotator-specific models and rapidly label large datasets, in an approach which has some similarities to transfer learning. As a baseline we completely removed any human labeling by labeling ROIs with pre-trained models using a consensus approach (Fig. 2b). By specifying the number of models needed for consensus we control how strict we wanted to be on what is considered a Pre-NFT/iNFT. This approach served to mimic consensus annotations by human experts/novices which has proven to be time consuming and logistically difficult to implement in practice.

Model-assisted-labeling can be used to leverage pre-trained models to create an initial set of labels for a dataset, at which point an expert can fine-tune for correctness. Labeling large datasets, such as those needed for this study, is very time consuming and exhaustive, and experts generally have neither the freedom nor desire to commit extensive effort to this process. Fine-tuning labels using a well-developed application, such as that which was developed and demonstrated in this work, substantially reduces annotation time and annotator fatigue without sacrificing performance [63, 64]. Furthermore, this workflow is generalizable; it is likely just as applicable to this kind of problem in the context of cancer as it has been shown to be in the case of neuropathology. Indeed, it may even transcend a single modality and prove efficacious in the context of ultrasound or MRI, for example.

In this study we showed model-assisted-labeling improves performance rapidly in the first few iterations but quickly plateaus (Additional file 3: Fig. S9). We implemented a “quantity over quality” approach, where we chose to incorporate a large number of ROIs with ML generated labels during training, adding additional ROIs with curated labels on each iteration. As would be expected, the best performing model was trained on only the set of ROIs with curated labels. While ML benefits from large quality datasets, and these often enable the model(s) to generalize well, simply feeding in large datasets with misleading or inaccurate labels can overwhelm the models and hamper learning (the well known “garbage in, garbage out” rule). Thus it is easy to conclude that for this neuropathology task, and indeed for these kinds of approaches in general, a “quality over quantity” mentality will almost always produce superior results.

In previous studies implementing ML methods for neuropathology tasks, we noticed little emphasis on workflow implementation in a “real-world” setting [36, 39]. WSIs are very large files with billions of pixels, and analysis on such a scale is an understandably daunting task, particularly for a human. Yet, while AI models can ingest images at a rapid pace not seen in most image analysis workflows, the sheer size of these images still means full-WSI analysis can take considerable time to accomplish. We address this challenge by leveraging the DSA infrastructure and the HistomicsUI viewer. HistomicsUI facilitates the visualization of, and interaction with, images and annotations inside an image viewer, and the DSA provides an application programming interface (API) that can be used directly from our AI workflow to interact with HistomicsUI bidirectionally. We utilized these tools to develop and implement a workflow which enables our models to predict NFTs on large regions (up to the entire tissue area for WSIs of any size) and display them in HistomicsUI for viewing. Our implementation showed a correlation between time taken to complete automated annotation and the relative abundance of the pathology of interest in the WSI. For example, WSIs with thousands of iNFTs/Pre-NFTs take considerably more time to complete than those with few. Annotating WSIs with little pathology can take less than 10 min to complete while WSIs with tens of thousands of Pre-NFT/iNFTs take closer to 40 min.

The workflow (referred to as inference workflow) contains several key steps, with the most time consuming step, other than prediction, being tiling/clean up. Tiling and clean up are necessary with the current implementation of the YOLOv5 AI model used, which requires us to save tiles locally for prediction, before then deleting. This is a time consuming process requiring input and output operations (I/O), which in newer versions of the YOLO model has been removed, and necessitates storage of duplicated data. The prediction step can be accelerated by adding additional resources to the workflow (in this case, GPUs were the primary bottleneck). In the case of this study, we utilized two GPUs at a time. Servers are limited to how many GPUs can be installed in them, generally two to four, but other software tools can be leveraged to implement AI inference across servers, such as NVIDIA’s Triton software (https://developer.nvidia.com/nvidia-triton-inference-server). While not tested, we could, in theory, even reduce time to process/predict on WSIs with tens of thousands of NFTs to just a few minutes. Much effort is currently being allocated to enable these kinds of inference workflows to run directly through HistomicsUI, further reducing the barrier to use for experts in domains other than computer science, AI, etc.

One additional benefit of implementing models using the inference workflows is the visualization of results at scale. Viewing results on the entire WSI, and indeed on multiple WSIs from a given case, or even a collection of cases, allows identification of patterns of NFT predictions, and facilitates the ability of experts to determine if they make sense in the context of what is already known about the particular disease or pathology (Figs. 5 and 6). Predictions in our workflow showed most NFTs are observed in the gray matter with only sporadic instances of them in the white matter, with most of those identified in white matter later confirmed to be false positives. This aligns well with what has been observed for this particular subset of neuropathology historically. Importantly, we were also able to identify three common mistakes made by our best model: (1) folding tissue being predicted as iNFTs, (2) edge staining being predicted as iNFTs or Pre-NFTs, and (3) non-NFT pathology, such as corpora amylacea, being predicted as iNFTs. Using HistomicsUI we were able to easily add new examples for these regions. This could be done rapidly since we were adding ROIs specifically with no NFTs, and did not require the time-consuming annotation step. This method proved to be effective at adjusting our models to avoid making mistakes mostly caused by image features the models did not see during training.

Imaging features from NFT ML detection have recently been shown to predict cognitive impairment, and similar detection of other tauopathies have shown to be predictive of disease diagnosis [38, 65]. In a similar approach, we showed we could use imaging features to accurately predict Braak NFT stages. Braak NFT staging displays inter-rater agreement and thus we reported our results in comparison to other expert raters [28]. Agreement from our predictor was good to excellent against a set of 5 raters, on a cohort of Emory cases. Limitations are present inherent to the rater being an ML model and not a human. The models are prone to a small level of false positives, which often lead to models not predicting Braak NFT stage 0 for any case (which usually display no tau pathology). This could also have been caused due to few cases of low Braak NFT stage being present during training. In this study, we collected a large dataset of labeled ROIs, but the number of available cases (n = 59) was small compared to what is normally recommended to train AI models. Regardless, we were able to create an open source passable ML Braak NFT stage rater that reported the same stages for a given case in our datasets. Additionally, this study provides an open-source dataset with human-level and machine-generated annotations of NFTs, which can be used in future studies.

Even so, this work strives to acknowledge the outstanding and unsolved challenges in the neuropathology field, where cohorts can vary considerably between institutions (Additional file 3: Fig. S11). Qualitatively we observed our best ML models could predict Pre-NFT/iNFTs on WSI from a different cohort—UC Davis. However, performance of our Braak NFT stage predictor was poor on a small cohort of UC Davis cases, and we hypothesize variations in the antibody between institutions may at least partially explain this difference. While the UC Davis cohort was entirely stained with the AT8 antibody, which has been reported to primarily stain Pre-NFTs in tissue, the Emory cohort was mostly PHF-1 stained, which primarily stains the iNFTs [8]. Yet further investigation into the subset of cases from Emory stained with AT8 did not show the expected similarity to the UC Davis cohort, suggesting other reasons, such as antibody dilution, incubation time, or even manufacturer, may be required to explain why NFT predictions on UC Davis WSIs were so markedly different.

Alternative approaches could be taken to tackle these challenges, with the simplest being to train the ML model on a UC Davis cohort and predicting on a separate UC Davis cohort. While Braak NFT staging ML model had poor performance, Pre-NFTs/iNFTs are predicted on the UC Davis cohort with good quality upon visual inspection. The difference appears to be what is actually on the images, thus training a model from a feature set extracted from UC Davis might predict Braak NFT stages comparable to humans on data from the same institution. In this study, we did not have the required amount of UC Davis cases to do this effectively, and future studies could attempt to do this on several cohorts from different institutions to validate this approach. A different approach might be taken though, where ML models are trained based on the antibody type used, which might be more translatable across centers with minimal work. This does of course pose its own sets of challenges, as recent published works have highlighted the amount of variability seen in antibody use across ADRCs and brain banks [31]. Creating a family of ML models that each capture a single antibody would be challenging, and first the validation of the YOLO NFT detection model would need to show robustness to various antibodies. A final and potentially the most robust approach, would be to extract more detailed features. The features used in this study are primarily based on the quantity of NFT type found in different regions, which as we show varies depending on antibody use/other features. Morphological features of NFTs (i.e. size, texture, key points such as SIFT, etc.) might be a more robust feature set that could translate across variations seen in cohort [66]

This workflow has potential far beyond neuropathology. Indeed we believe it can provide utility in the various pathology subfields, such as cancer and nephrology, and even fields as far flung as Astronomy, which similarly processes exceptionally large and complex imaging data [67, 68]. Though even just within neuropathology, this work is likely only the beginning, and we hope the dataset generated and discussed herein, which has been made public, will be the foundation from which many workflows may be created. Future work will focus on using transfer learning to allow detection of more diverse neuropathology relevant features such as neurons, pTDP-43 inclusions, and Lewy bodies.

Understanding the relationship between imaging features, biomarkers from multi-omics approaches, and clinical data is of increasing importance and is being discussed with great urgency, particularly in the context of highly complex, poorly understood diseases [69]. We envision the possibility of new ways of phenotyping neurodegenerative disease, by creating deep imaging features to describe neuropathology cases with a granularity not currently possible. Finally, we intend to provide this workflow in an accessible format via the DSA/HistomicsUI, in hopes that we may aid the field of neuropathology, and those practicing it, in more readily meeting the challenges of the data-driven future of this domain.

## Conclusions

In this study we tackled the task of NFT detection in WSIs and Braak NFT staging of patient tissues using a supervised learning approach with object detection models. We demonstrated the complexity of annotating these tissues for neuropathology inclusions and the tendency for poor inter-annotator agreement. Leveraging a model-assisted-labeling approach, we show the relative ease with which models may be improved by artificially labeling a larger set of images on a diverse cohort of Emory cases, without the need of expert knowledge initially. These models were then used in a novel workflow that efficiently identifies Pre-NFT & iNFTs within entire WSIs, and extracts a set of features that describes each case. These features, alongside an ML model, successfully predict Braak NFT stages for Emory cases, displaying high agreement with experts. Implementing the reported approach with a cohort sourced from another institution showed that staining variations, potentially due to differences in immunohistochemical antibodies, leads to different results between institutions. This study integrated AI workflows with an image viewer that is accessible to experts in neuropathology, with our goal being to show ML can provide practical utility for the field, and to demonstrate a workflow which facilitates this. Future work will aim to integrate the workflow (Pre-NFT/iNFT detection, model-assisted-labeling, and automated NFT Braak staging) developed in this study into a usable interface, making it easy for persons to use routinely and at scale.

### Supplementary Information

**Additional file 1**. Spreadsheet with metadata for all WSIs used in this project.

**Additional file 2**. Original instructional document provided to raters/annotators, includes guidelines to use when providing Braak NFT stages and Pre-NFT/iNFT annotations.

**Additional file 3**. Supplementary Figures, Tables, and sections for additional information.

## Data Availability

The images (regions of interests) used to train and evaluate all machine learning models as well as data created and used in this project, including results, are available at https://drive.google.com/drive/folders/16LUMrIMdp4LlvWQk5Dp3eVQHWY472jN5?usp=sharing. WSIs are available for download upon request, please contact David Gutman. The Python scripts, Jupyter notebooks, and additional code information are available at https://github.com/Gutman-Lab/yolo-braak-stage. The README.md file contains detailed information on each file and project setup.

## Abbreviations

ML: Machine learning
NFT: Neurofibrillary tangle
WSI: Whole-slide-image
Pre-NFT: Pre-tangle stage of NFT
iNFT: Intraneuronal NFT
AD: Alzheimer disease
aβ: Amyloid beta
NIA-AA: National Institute on Aging-Alzheimer’s Association
YOLO: You only look once model
ADRC: Alzheimers Disease Research Center
ROI: Region of interest
GPU: Graphical processing unit
IoU: Intersection over union
DSA: Digital slide archive
FOV: Field of view
