# Supplementary material for: Toward a generalizable machine learning workflow for neurodegenerative disease staging with focus on neurofibrillary tangles
Source: Acta Neuropathol Commun. 2023 Dec 18;11:202. doi: 10.1186/s40478-023-01691-x (PMC10726581; doi:10.1186/s40478-023-01691-x)
Supplement: Supplementary file 2 — Additional file 2. Original instructional document provided to raters/annotators, includes guidelines to use when providing Braak NFT stages and Pre-NFT/iNFT annotations. [file 40478_2023_1691_MOESM2_ESM.docx]

Braak Staging and NFT Criteria

The protocol in this study calls for (1) providing Braak stages and (2) annotating intraneuronal NFTs (iNFTs) and pre-NFTs on tau immunostained whole-slide images (WSIs). Braak staging will be done using four brain regions: hippocampus, temporal cortex, occipital cortex, and amygdala.

Following is some further information about the region slides in the Emory cohort.

- Hippocampus: mostly posterior hippocampus, the slide may contain the left or right hippocampus or both. In a very few cases there will be both a left and right hippocampal slide, both are provided and can be used in the Braak staging.
- Amygdala: this serves as the anterior hippocampus that the Braak staging protocol uses in Braak et al. 2006: <https://link-springer-com.proxy.library.emory.edu/article/10.1007/s00401-006-0127-z>.

# (1) Braak Stages - expert annotators only

Follow the Braak staging criteria defined in Braak et al. 2006 (Staging of Alzheimer disease-associated neurofibrillary pathology using paraffin sections and immunocytochemistry, <https://link-springer-com.proxy.library.emory.edu/article/10.1007/s00401-006-0127-z>)

Follow page 396 (6 of article) and associated figure 4.

The paper does not quantifiably define low, moderate, or severe amounts of NFTs in a certain region which is used in the wording of the Braak staging instructions. This is left to the interpretation of each annotator in the Braak staging protocol.

# (2) NFTs annotations - expert & novice annotators


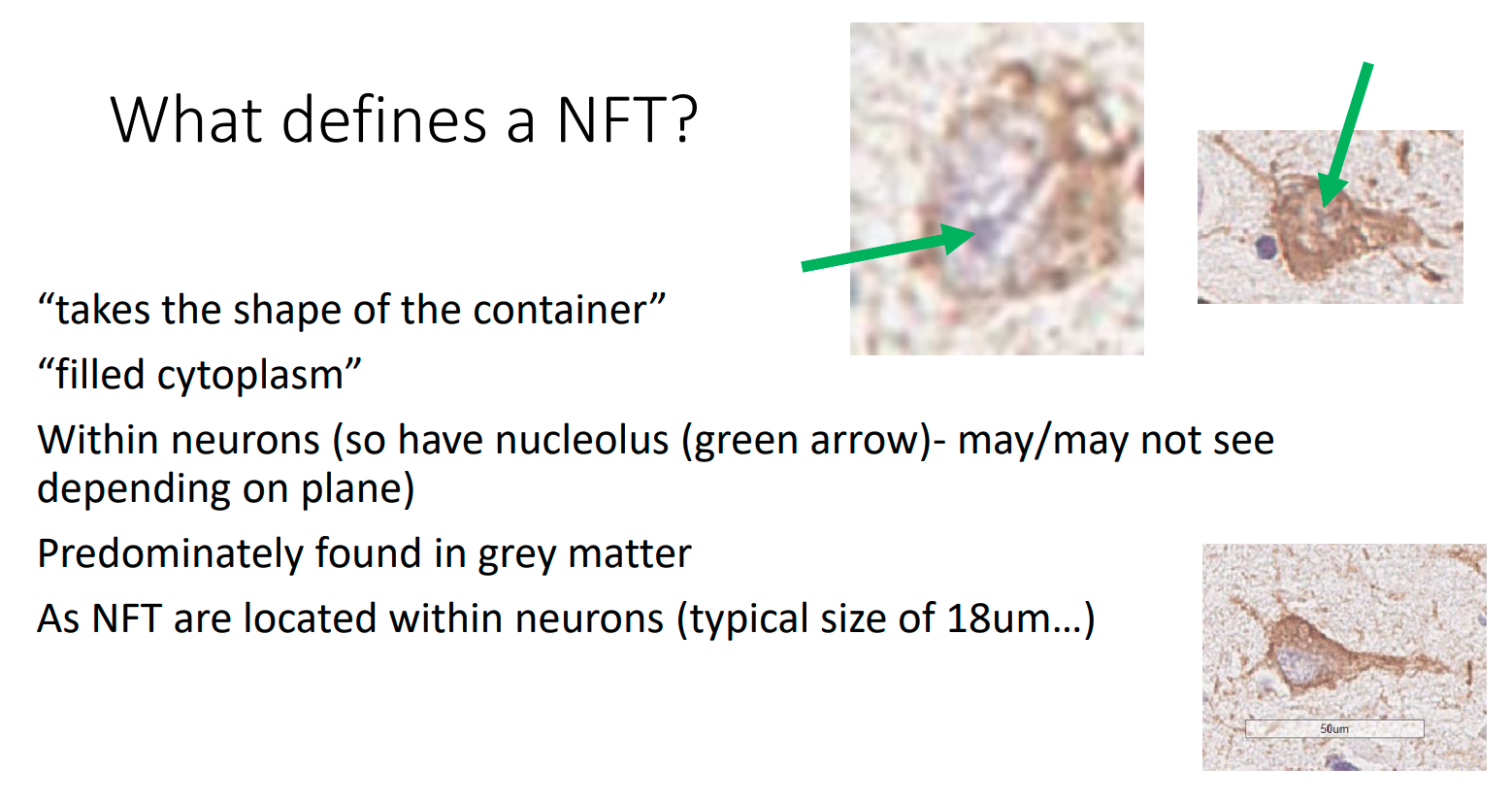


Definition of a neurofibrillary tangle (NFT) - taken from Dr. Brittany Dugger’s slides.

Pathologists and novices will provide point annotations for two classes of NFTs - intraneuronal NFTs (iNFTs) and pre-NFTs. The HistomicsUI annotation system will be used to drop a point annotation (done by left click of the computer mouse) **near the center of the NFTs body** (not the processes) while taking care to select the correct annotation label (iNFT or Pre-NFT). ROI boxes have been assigned to each annotator in the WSIs (same WSIs used for Braak staging) which they should exhaustively annotate for iNFTs and pre-NFTs.


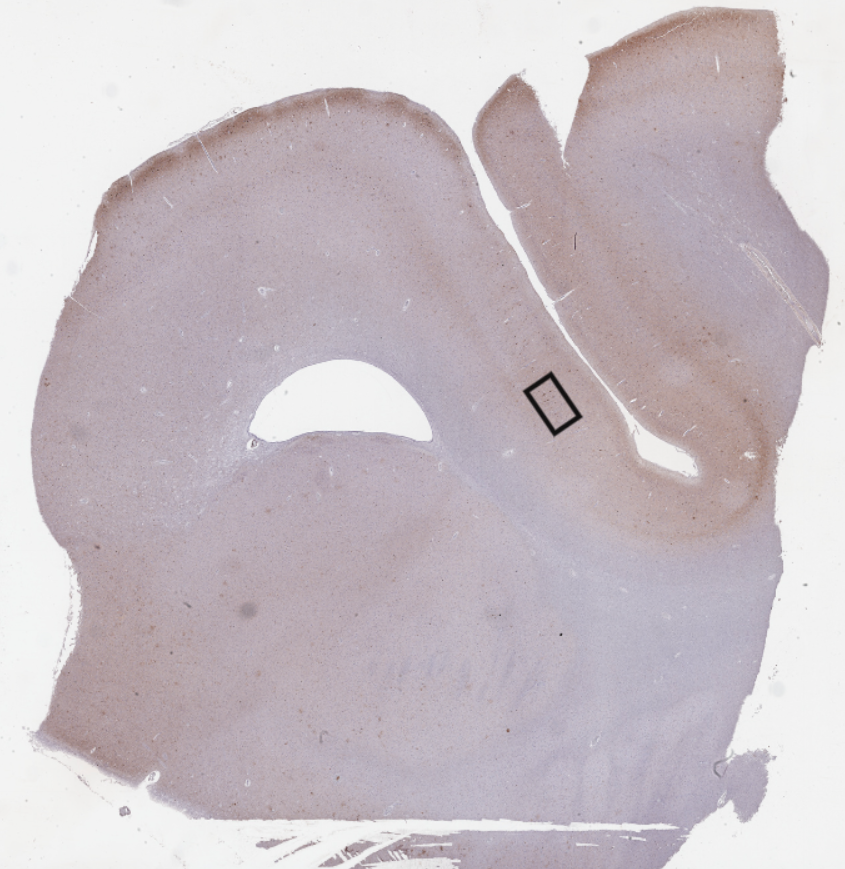


A WSI showing a box ROI annotation. Annotators should exhaustively annotate these ROIs for iNFTs and pre-NFTs.

Following are detailed descriptions of what defines an iNFT and pre-NFT in our protocol that should be taken into account when annotating the ROIs. This information was created using advice from Dr. Crary and Augustinack *et. al.* 2002 (Acta Neuropathol, <https://link-springer-com.proxy.library.emory.edu/article/10.1007/s004010100423>).

**iNFT**

iNFTs should be a dark brown color, taking into account the hue of the particular slide you are looking at


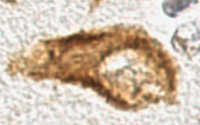

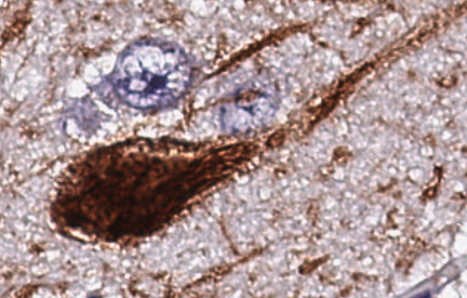


These two are both iNFTs but the hue of each WSI is different. The iNFT on the right looks much darker than on the left but also the background staining is much stronger on the right.

The inclusion should be fibrillary - hair like in texture - but densely packed and homogenous (i.e. not broken up or punctate)

iNFTs may have the nucleus displaced to the side of the cytoplasm


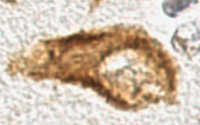


Fibrillary texture is seen in this iNFT. Also note that the nucleus is visible and pushed to the side.

Phospho-tau staining will often be seen in the dendrites and the axon hillock


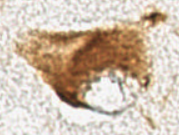


Staining is seen in the axon hillock and some processes are also visible (top right).

Dendrites may appear deteriorated

In some cases the nucleus may not be visible at all but if the brown is dark and fibrillary and clearly has the shape of a dendrosomatic compartment (soma plus dendrites and axon hillock) it can be counted as an iNFT.


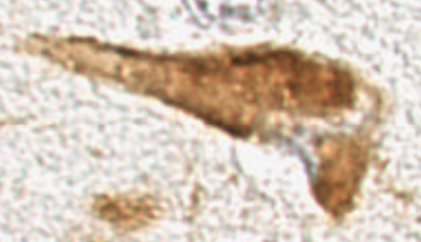

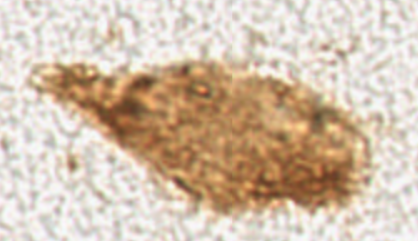

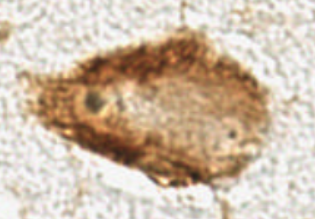


Examples of iNFTs where the nucleus is barely visible or not visible at all but the inclusion has a clear dendrosomatic compartment.

Note that neuron nucleus is ~10 µm and the iNFT will be slightly larger ~15-20 µm


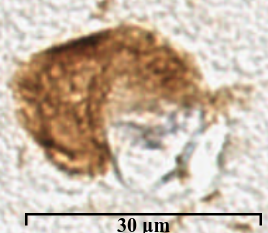


Note - extracellular NFTs may appear similar to iNFTs but show few discernable dendrites and no visible nucleus, the compartment also looks less like a cell containing compartment. These should not be counted as an iNFT.


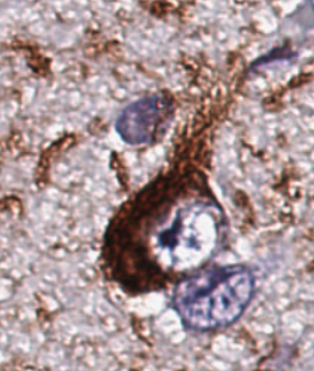

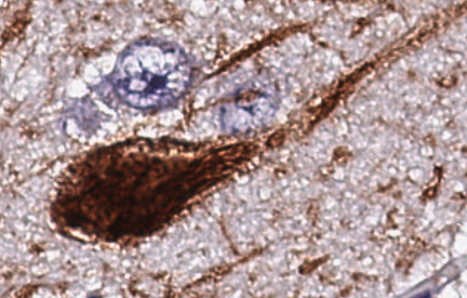


*Left:* An iNFT which shows the nucleus pushed to the side of the cell and the tau inclusion surrounding mostly one side. You can also see the tau inclusion in what looks like the axon (top right). *Right:* A clear iNFT that does not show a nucleus. The shape of the tau inclusion is a clear dendrosomatic compartment with an axon tail clearly visible. Notice that both inclusions have a hair-like texture, this is a common feature in both iNFTs and pre-NFTs but are much more densely packed in iNFTs.

**Pre-NFT**

Has a lighter brown color surrounding a nucleus with less packed fibrillary texture

A nucleus should be clearly visible to be counted and may even display a clear nucleolus (visible nucleolus is not a requirement)

May have a small punctate region (i.e. not a continuous brown positive region, may be broken up). Note that there are occasions when other types of inclusions might be present that disrupt the look of an NFT, such as granulovacuolar degeneration and Lewy bodies.


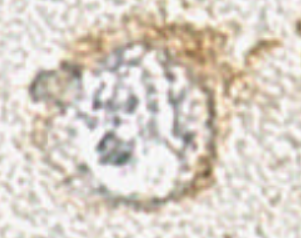
**
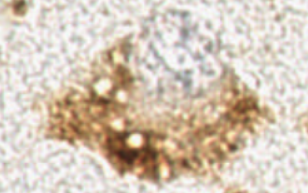

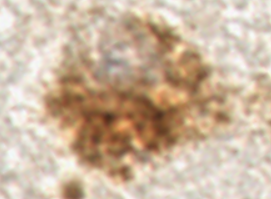
**

Examples of pre-NFTs that show clear nucleus and much less-fibrillary and dark tau staining. Notice on the middle image you see little empty vacuoles which is a sign of granulovacuolar degeneration - these types of other pathology should be kept in mind as they may influence the decision of what type of NFT it is. The right image also shows a nucleus but it is less hard to see, these should still be counted as pre-NFTs.

Dendrites can be clearly visible and axon tail may be visibly filled with tau and some processes visible


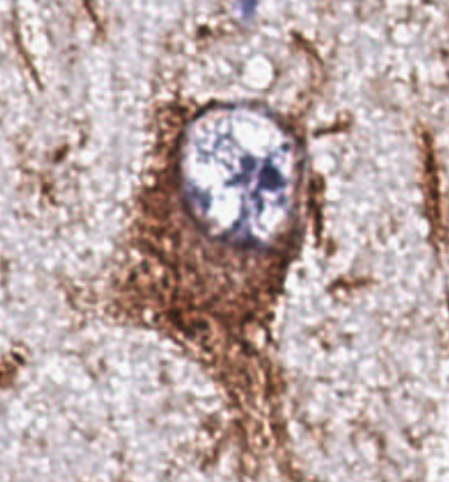

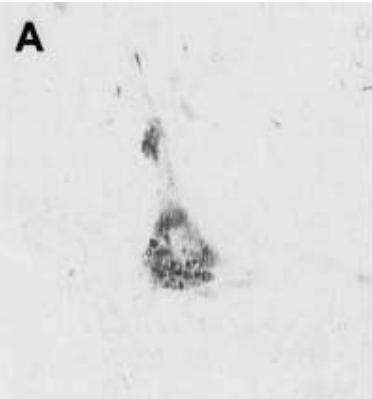


*Left:* An example of a pre-NFT which shows lighter brown fibrillary texture and a clear nucleus being surrounded. Note that this also has the nucleus pushed to the edge of the neuron and a clear axon tail. *Right*: an example of a pre-NFT viewed with TG3 antibody showing the punctate phospho-tau positive staining (dark). Figure taken from Augustinack *et. al.* 2002 (Acta Neuropathol).
