# Supplementary material for: Toward a generalizable machine learning workflow for neurodegenerative disease staging with focus on neurofibrillary tangles
Source: Acta Neuropathol Commun. 2023 Dec 18;11:202. doi: 10.1186/s40478-023-01691-x (PMC10726581; doi:10.1186/s40478-023-01691-x)
Supplement: Supplementary file 3 — Additional file 3. Supplementary Figures, Tables, and sections for additional information. [file 40478_2023_1691_MOESM3_ESM.docx]

# Codebase & Data

All our code is published in the GitHub repository: <https://github.com/Gutman-Lab/yolo-braak-stage>.

We utilized the Digital Slide Archive (DSA) throughout this project, and all the scripts we developed integrate with the DSA when pushing annotations or metadata back and forth. However, because of this not all scripts will be deployable by everyone. We are not providing access to the DSA instance with the WSIs, to avoid repeated calls modifying the annotations we have collected and stored in the DSA / HistomicsUI viewer. We instead are providing access to all our data generated from the scripts for open-source download and a zip file containing just the ROI images and best labels for these. Link to data downloads is: <https://drive.google.com/drive/folders/16LUMrIMdp4LlvWQk5Dp3eVQHWY472jN5?usp=sharing>

Additionally, in the GitHub repository we provide tutorial Jupyter notebooks with examples for:

1. Tiling ROIs with labels
2. Created dataset YAML files for training YOLOv5 models
3. Training YOLOv5 models
4. Inference
   1. Inference on an ROI level
   2. Inference on entire WSIs

# YOLO Models

YOLO models were trained for detection of Pre-NFTs and iNFTs. We used a forked version (<https://github.com/jvizcar/nft-detection-yolov5>) of Ultralytics’ YOLOv5 repository (<https://github.com/ultralytics/yolov5>). Modifications to the source repository were implemented to be more appropriate to this project. Data augmentation workflow in the repository allows transformations, such as translation and rotation, that will partially cut off NFT boxes. The default parameters will still keep a box as a positive label if at least 10% of the original box is still present. For NFTs, only having 10% of an NFT in the image is not sufficient to differentiate from background staining. This default setting was changed to 50%, to improve performance. Similarly, the default YOLO approach is to use non-max suppression (NMS) to remove redundant boxes in an non-agnostic manner. This means that each class of boxes is treated separately, and thus multiple boxes can overlap considerably so long as they have different labels. For our task this does not make sense, since a Pre-NFT can’t also be an iNFT - they are two different and mutually exclusive classes. Thus, the second change to the repository was to use agnostic based NMS during training and validation of the datasets.

Hyperparameter tuning for YOLO models was done manually, using Weights and Biases (<https://wandb.ai/>) for visualization of training runs. We used a subset of various annotator datasets to train models with different hyperparameters, and averaged results to compare different parameters. The object (0.7), class (0.3), and box (0.05) loss gains were modified initially to prevent large fluctuations in losses during training epochs. All other parameters of the models were kept to the default values. The YOLO models used stochastic gradient descent with a learning rate of 0.01.

Data augmentation used during all runs were also tuned in a similar fashion and also incorporated domain knowledge to use appropriate transformation. Color augmentation was kept to a minimum since brown is a strong feature of detection of NFTs. The biggest change was the removal of mosaic data augmentation, which stitches multiple images together into a mosaic during training. We identified this as being detrimental to our model performance and thus removed it from all training runs.

All annotator models were trained to 100 epochs, with early stopping occurring after 20 epochs of no improvement on the validation dataset. The batch size was set to 24, but we used two GPUs for training, without batch normalization. Thus each batch contained 12 images per GPU. All model training started from COCO trained weights and used the YOLOv5m6 architecture. For training the larger models, containing all 497 ROIs, we trained to 150 epochs with the same early stopping criteria.


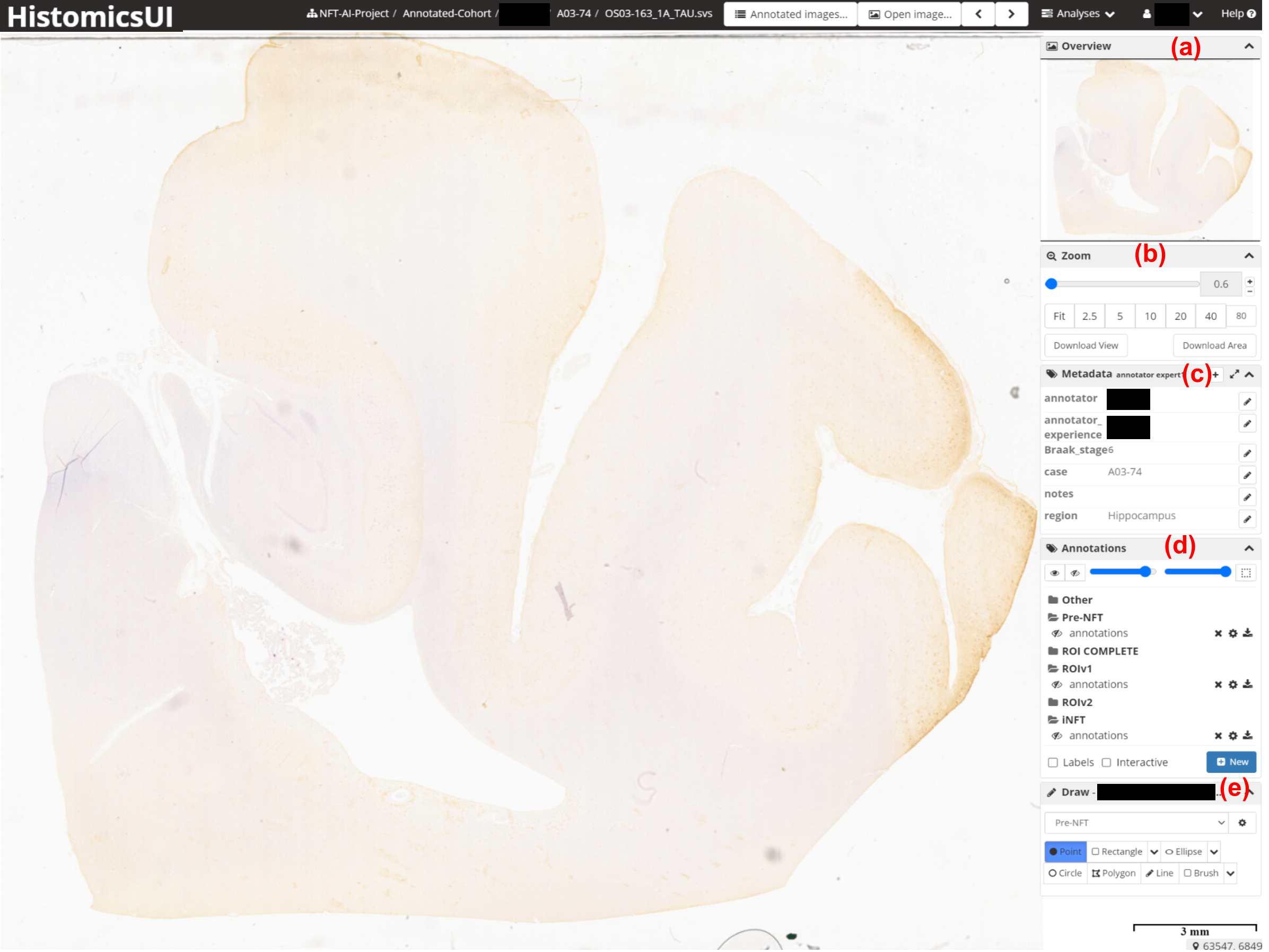


**Supplementary Fig. S1. HistomicsUI viewer.** The top banner displays the name of the WSI and its location in the file system, and can be used to log in using unique user credentials, navigate between WSIs (“Open Image” button) available, and access the help page. Additional menus are provided on the right, all are collapsable to remove clutter. The “Overview” menu **(a)** allows quick navigation around the WSI. The “Zoom” menu **(b)** allows controlling the magnification, but this can also be accomplished using keyboard shortcuts and the mouse wheel. The “Metadata” menu **(c)** allows modification of metadata associated with the current WSI (including adding and deleting metadata). The “Annotations” **(d)** and “Draw” **(e)** menus allows toggling visibility of annotations and adding new annotations. The “Draw” menu provides various annotation types to choose from, of interest in this project are the “Point” and “Rectangle” type annotations. The “Help” link provides access to detailed documentation on how to use HistomicsUI, including advanced tips on useful keyboard shortcuts and annotation tricks.

**
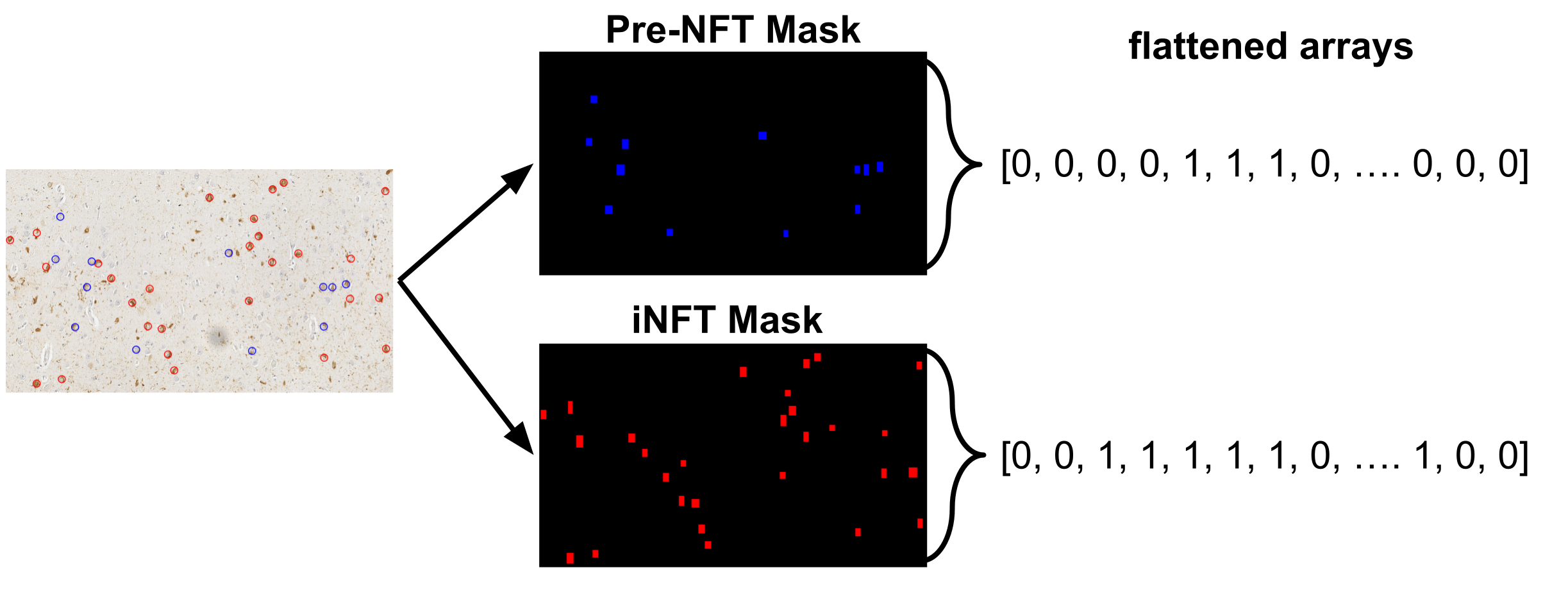
**

**Supplementary Fig. S2.** **Process used to calculate Cohen’s kappa for Pre-NFT / iNFT annotations.** An annotated ROI is shown on the left, with red circles representing iNFTs and blue circles representing Pre-NFTs. These annotations contain single points, shown as circles for visualization. The point annotations are converted into bounding boxes using a custom watershed approach followed by a manual quality check step.The bounding boxes are used to create label mask for each class of inclusion (middle images). The label masks are then flattened into a vector where 0 represents a pixel with no Pre-NFT / iNFT present and 1 represents a pixel with Pre-NFT / iNFT present. These vectors or flattened arrays are used to calculate the Cohen’s kappa for pairs of annotators. Cohen's kappas are calculated for each ROI, and then averaged for pairs of annotators. We analyze the two classes of objects separately for this analysis.


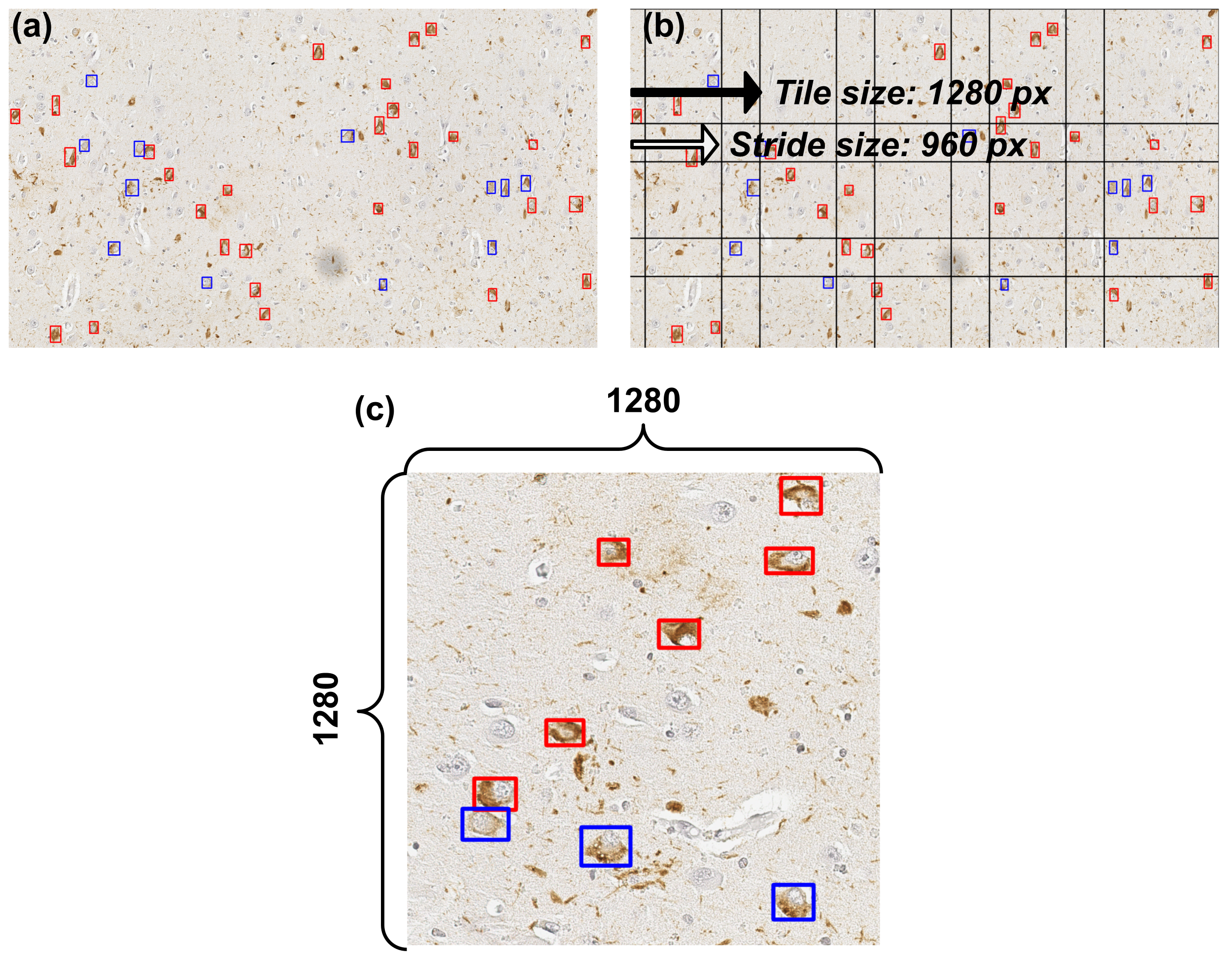


**Supplementary Fig. S3. Example of a region of interest (ROI) with Pre-NFT (blue) and iNFT (red) box annotations drawn for visualization. (a)** ROIs are typically several thousand pixels in width and height, too large to use efficiently when training modern AI models. **(b)** The ROIs are divided into a smaller set of images by tiling, or splitting the image via a grid pattern, with each tile image being 1280 x 1280 pixels (solid arrow). We use a stride of 960 pixels between each tile so there is image overlap between adjacent tiles (i.e. parts of the ROI image appear in multiple tiles, clear arrow). **(c)** A tile image is shown, with its dimensions and for visualization the box annotations for the Pre-NFT / iNFT boundaries. px = image pixels. Boxes partially cut off by the tiling process are kept if at least 50% by area is in the tile.


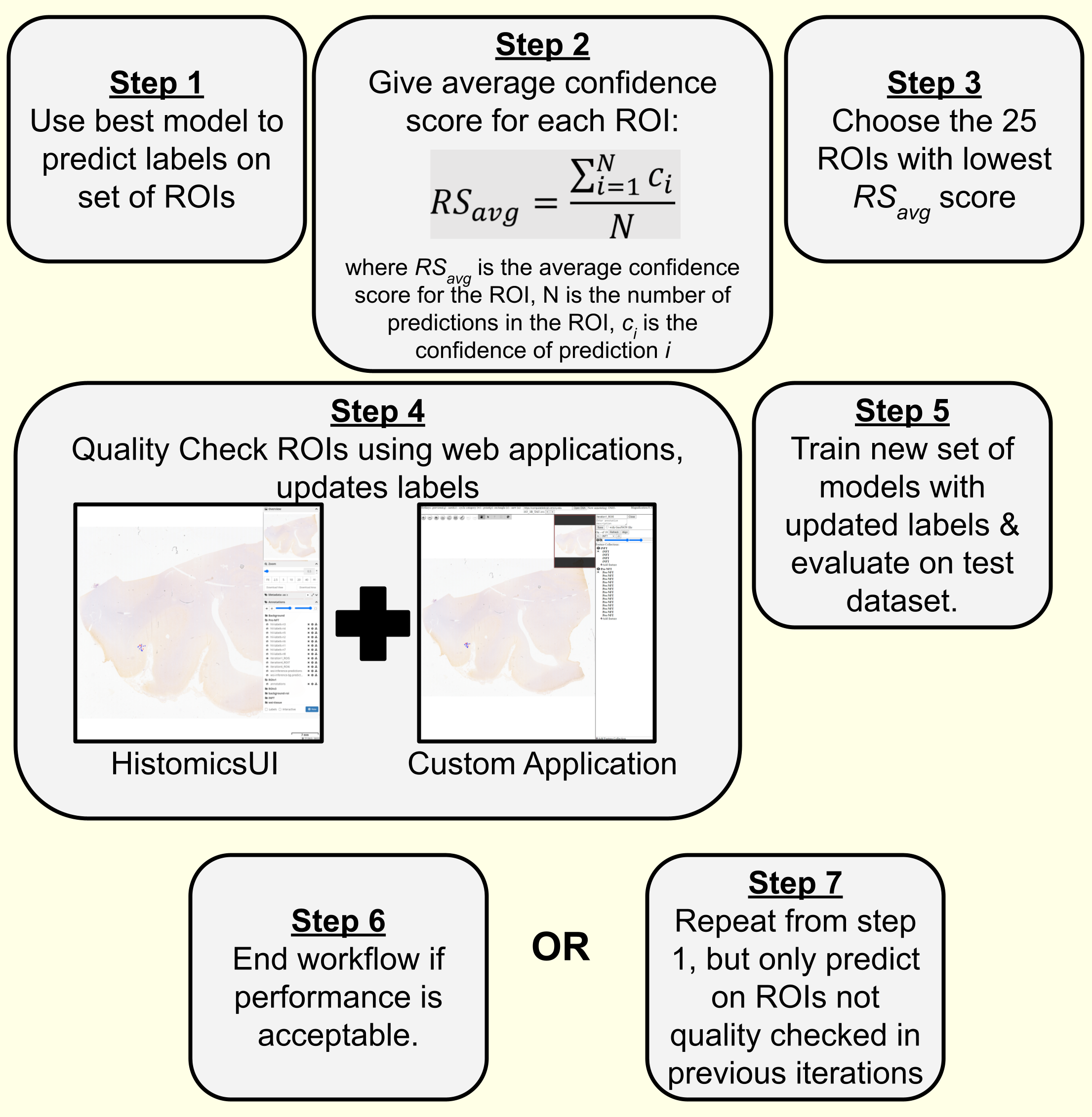


**Supplementary Fig. S4. Workflow for model-assisted-labeling.** The custom web application adds keyboard shortcuts for cycling between predictions rapidly, changing the label between Pre-NFT / iNFT, and deleting false predictions. HistomicsUI is used to then pan around the ROI and add labels when they are missed.


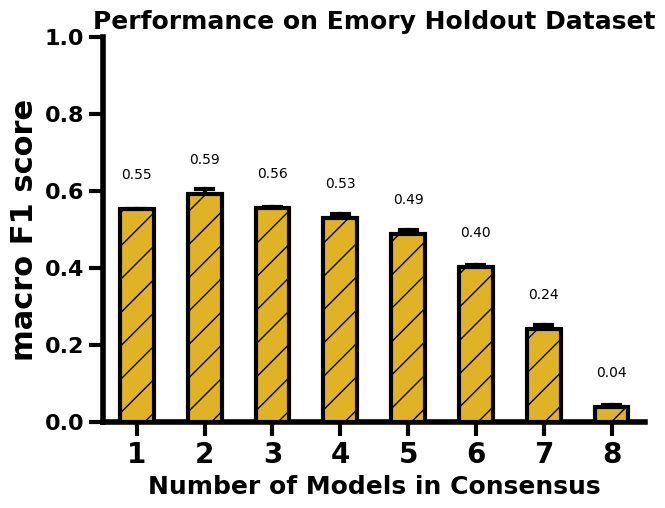


**Supplementary Fig. S5.** **Performance of models trained on a large dataset annotated by consensus of pre-trained model predictions.** The models are evaluated on the Emory-Holdout dataset and the average of three-fold cross-validation models are shown with error bars representing the standard deviation.


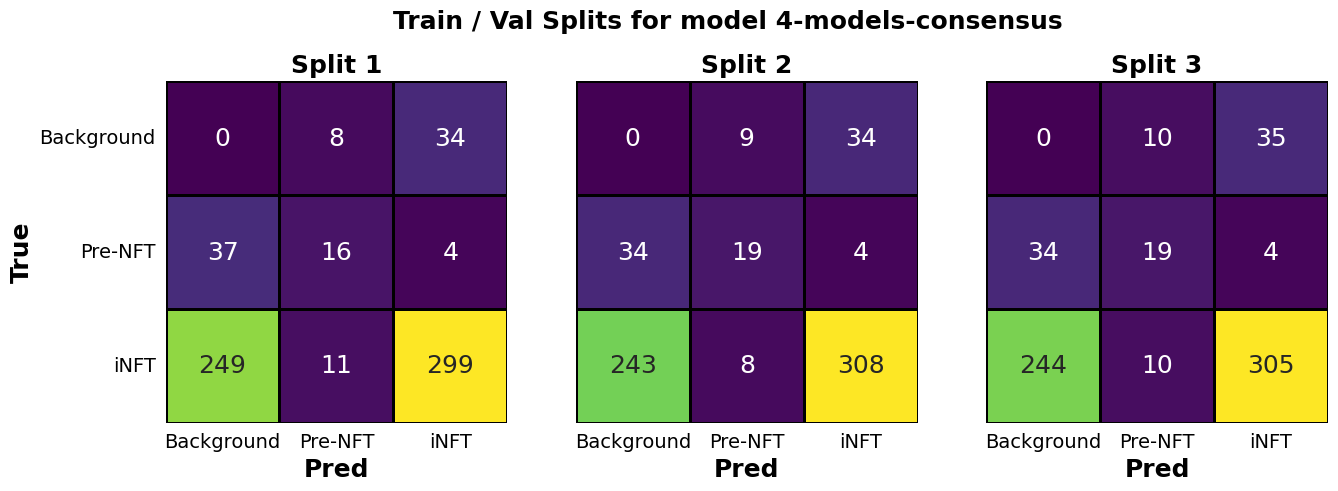


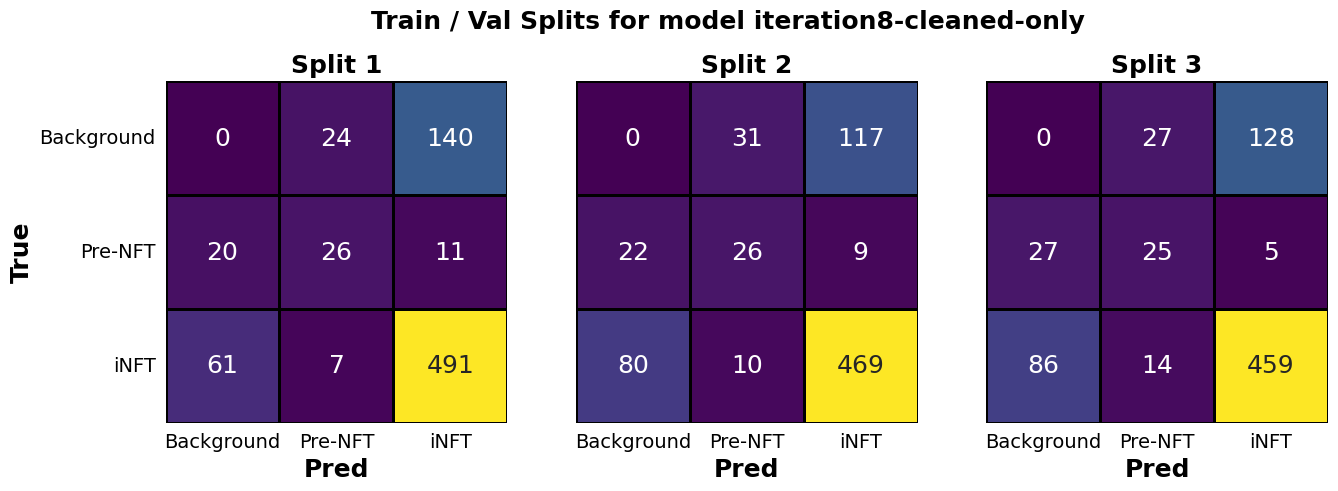


**Supplementary Fig. S6.** **Confusion matrices of the YOLO models for the base consensus models using model-assisted-labeling and the final model after 8 iterations (only the curated ROIs).** The model improved and the type of errors made changed from mostly false negatives to False positives for iNFT class. The three splits represent the three-fold cross-validation models.


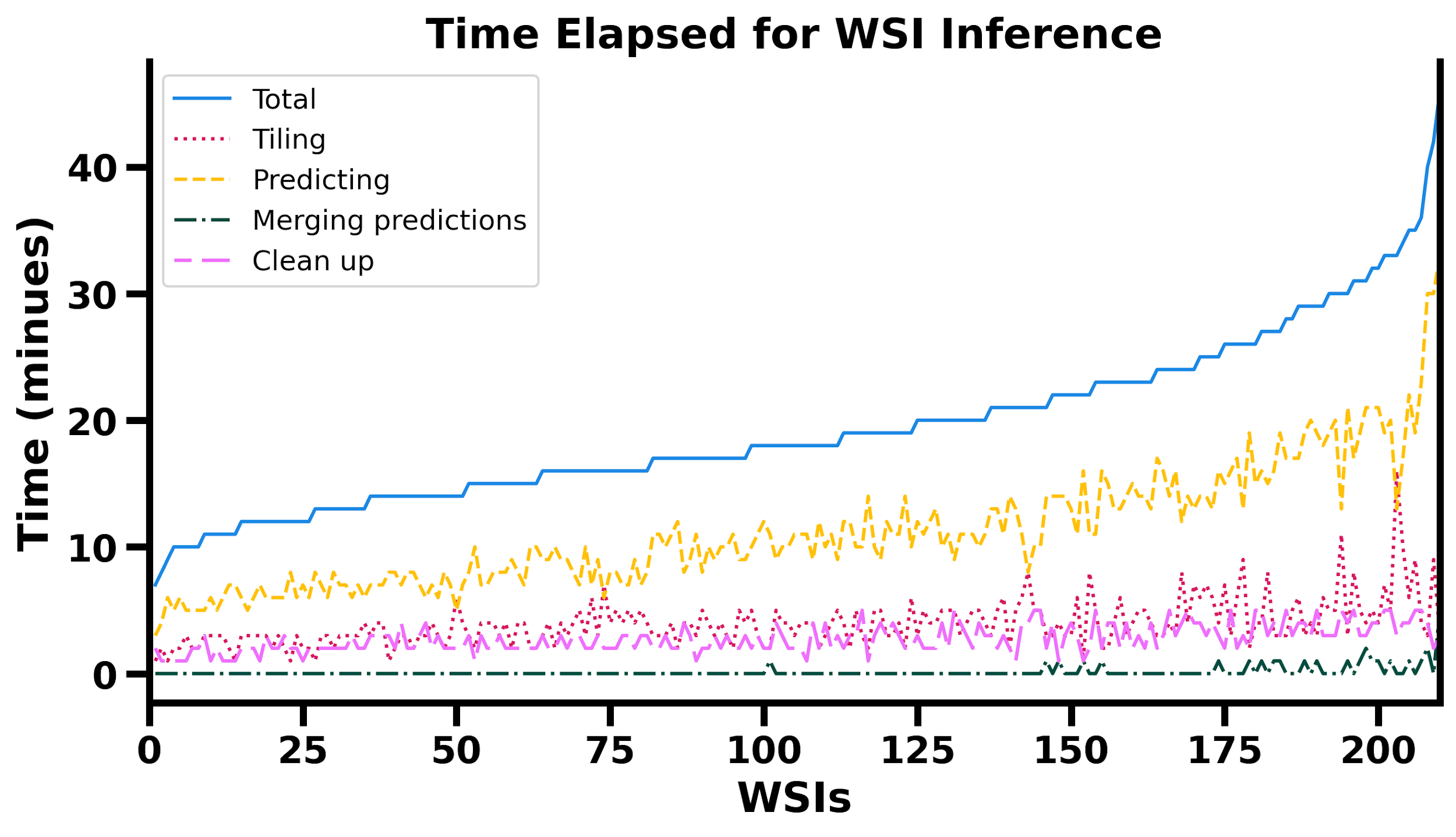


**Supplementary Fig. S7. Inference time for predicting Pre-NFTs / iNFTs in WSIs, for the 52 case Emory-Train cohort.** The total time varies considerably with some WSIs taking less than 10 minutes while a few take over 40 minutes. The most time consuming operation is the predicting on tiles / patches. Tiling and deleting the tiles after completion also takes several minutes but is mostly consistent between WSIs. The merging of overlapping predictions is highly optimized and is a non-limiting factor.


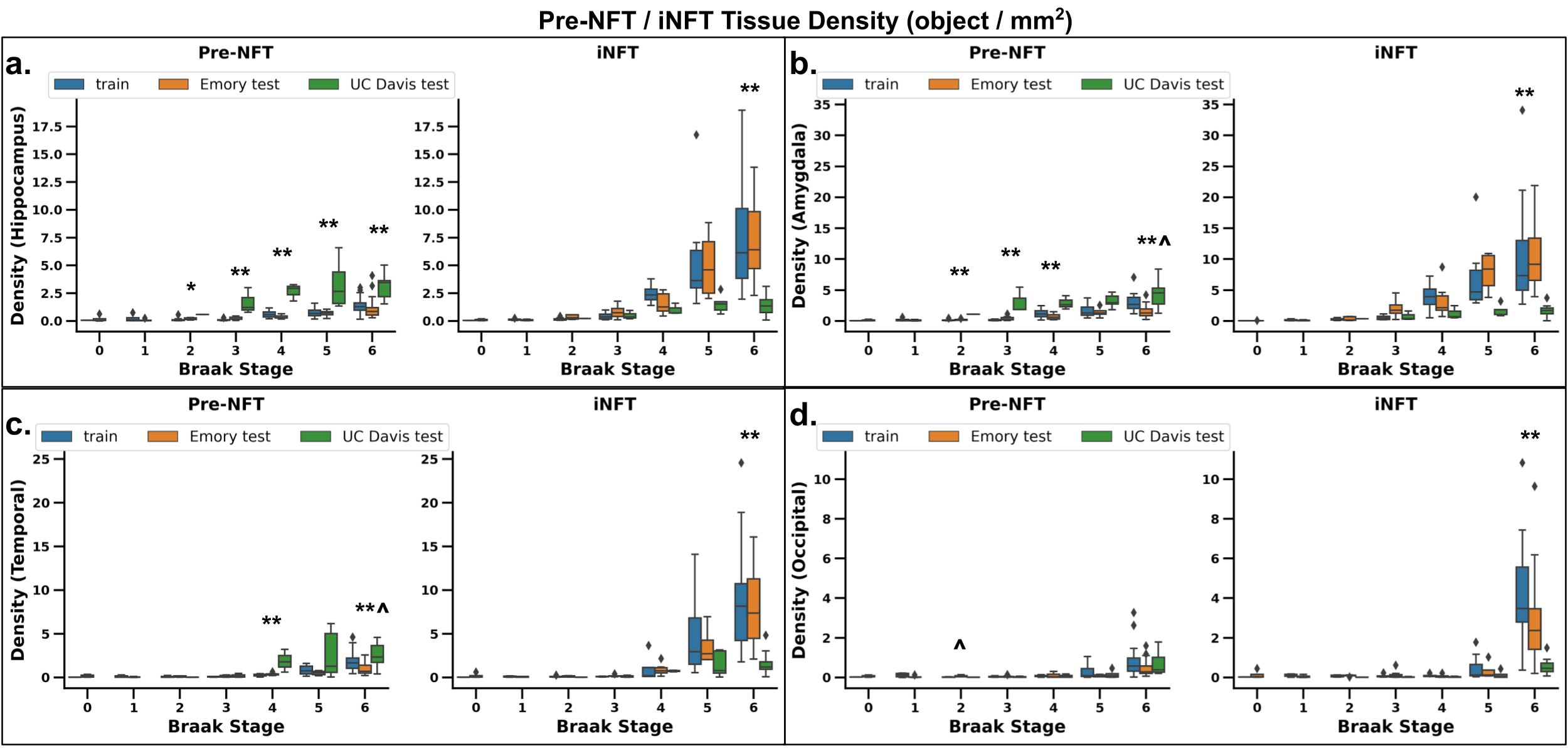


**Supplementary Fig. S8. Density of Pre-NFT and iNFT objects in the brain tissue by region and cohort.** In the hippocampus (a) and amygdala (b), signal is observed starting in early stages (>I) while signal is only observed in later stages (>III) in the temporal (c) and occipital cortex (d). In the Emory cohorts we also see higher densities of iNFTs compared to Pre-NFTs in all regions and Braak NFT stage groups. In the UC Davis cohort we see the reverse of this, where Pre-NFTs have a higher tissue density than iNFTs in the same regions. Diamonds represent outlier points that are more than 1.5 times the interquartile range of the data. Statistical significance was measured in each Braak NFT stage group between the cohorts using an one-way ANOVA with a significance value of 0.05. If significance was observed a post-hoc Tukey’s test was applied to identify which groups are significantly different. * UC Davis cohort is statistically different from one Emory cohort, ** UC Davis cohort is statistically different from both Emory cohorts, ^ Emory cohorts statistically different from each other.


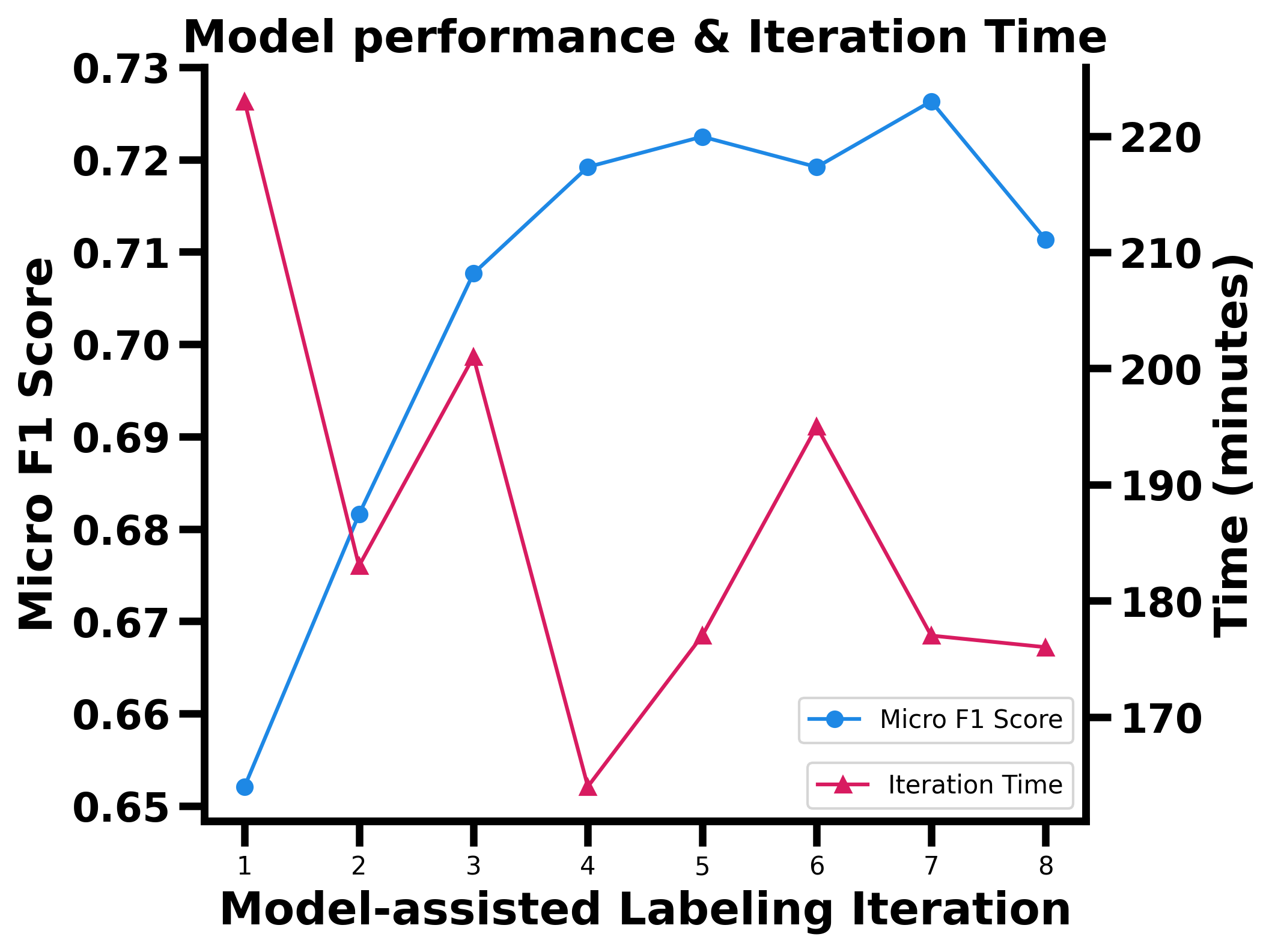


**Supplementary Fig. S9. Performance and time for each iteration of model assisted labeling.** Performance increases substantially initially but eventually plateaus in the end. Time taken per iteration, in minutes, varies considerably but was highest on the first iteration.

**
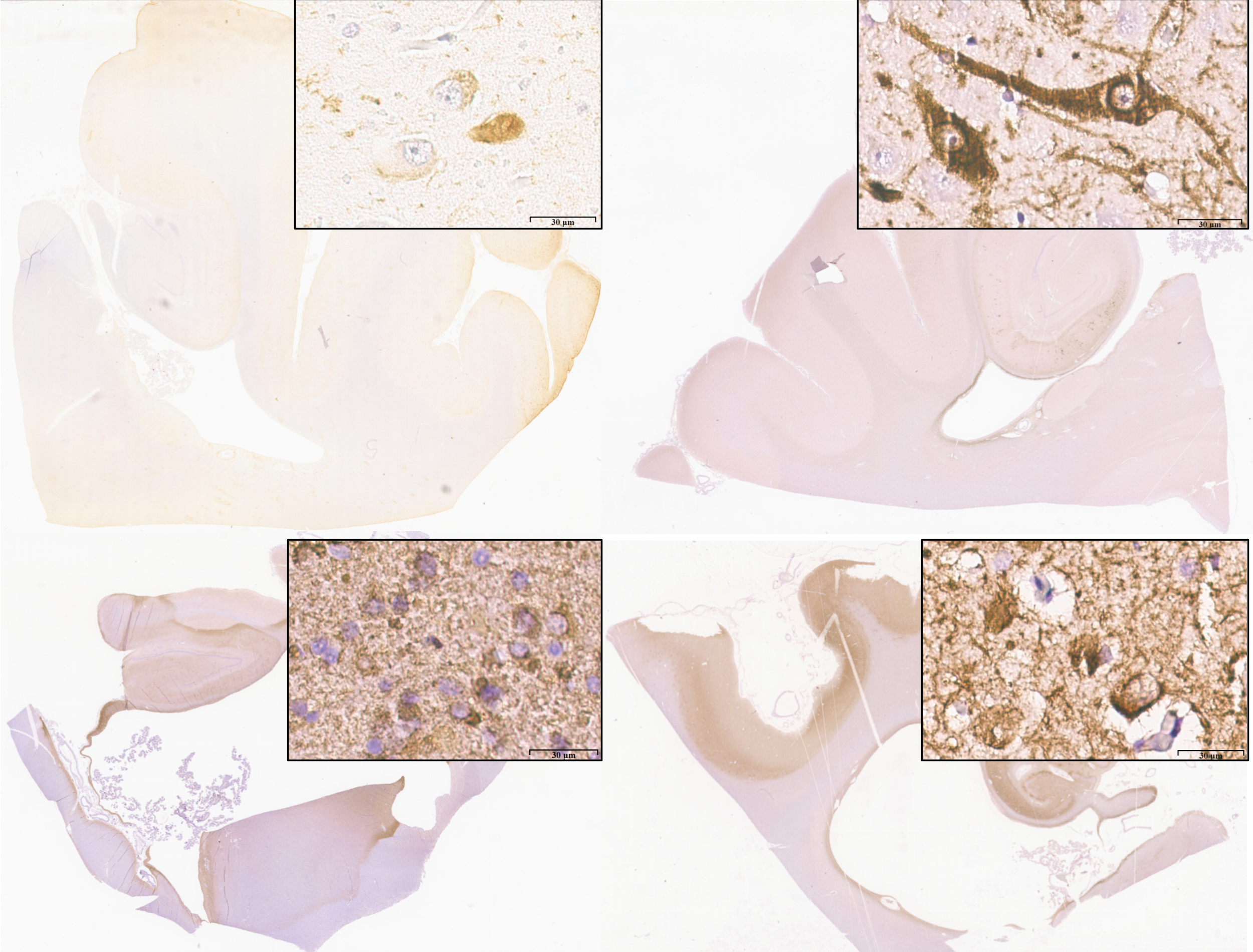
**

**Supplementary Fig. S10.**  **Examples of low background staining WSIs (top row) vs high background staining WSIs (bottom rows).** Background staining can be caused by differences in staining procedures followed, quantity of tau pathology present (neuropil threads are a primary source of background staining in cases with heavy tau pathology), age of slide at the time of digitization, etc. Insets show high resolution sections of the WSI.

**Emory Case**

**
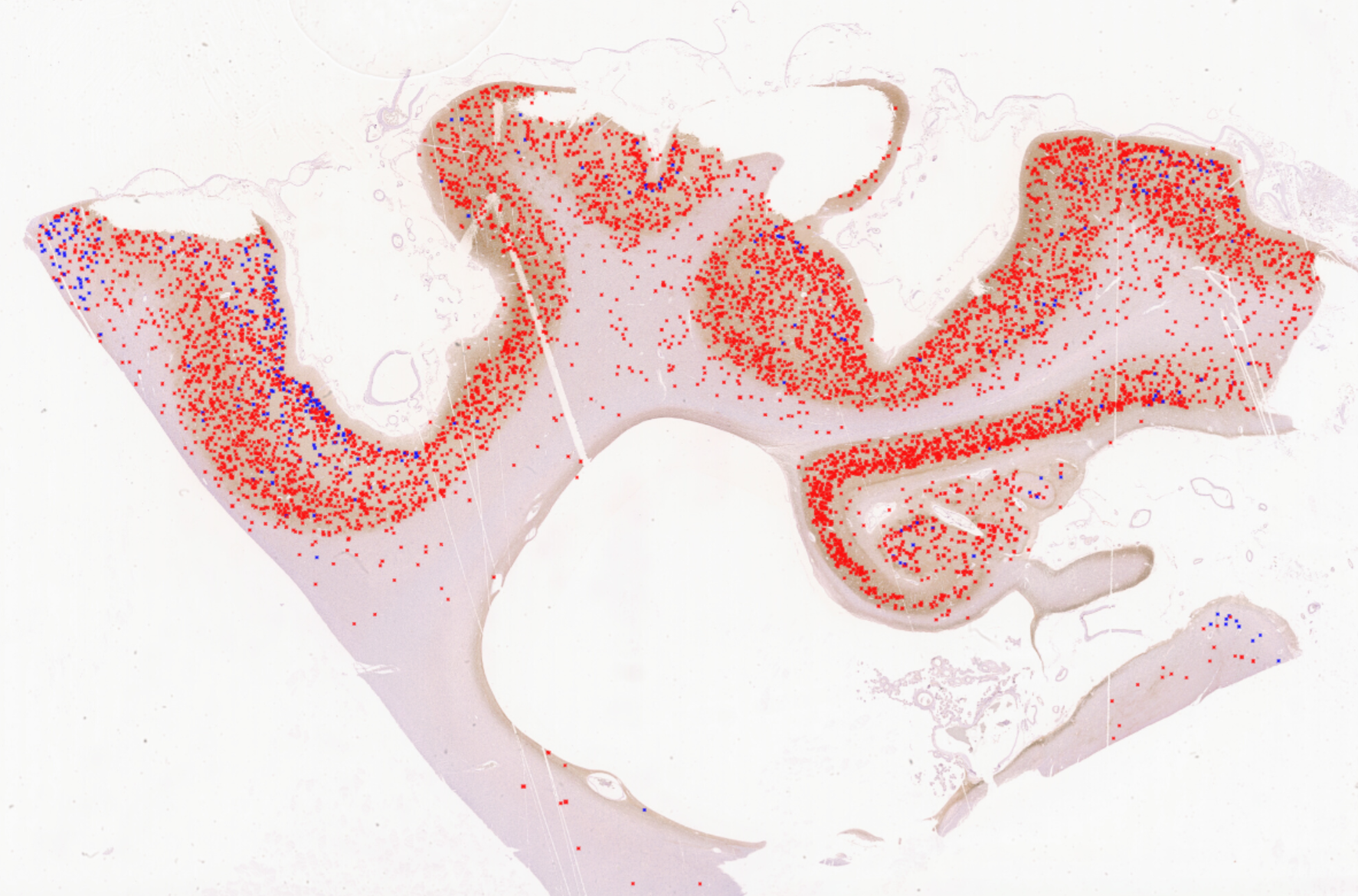

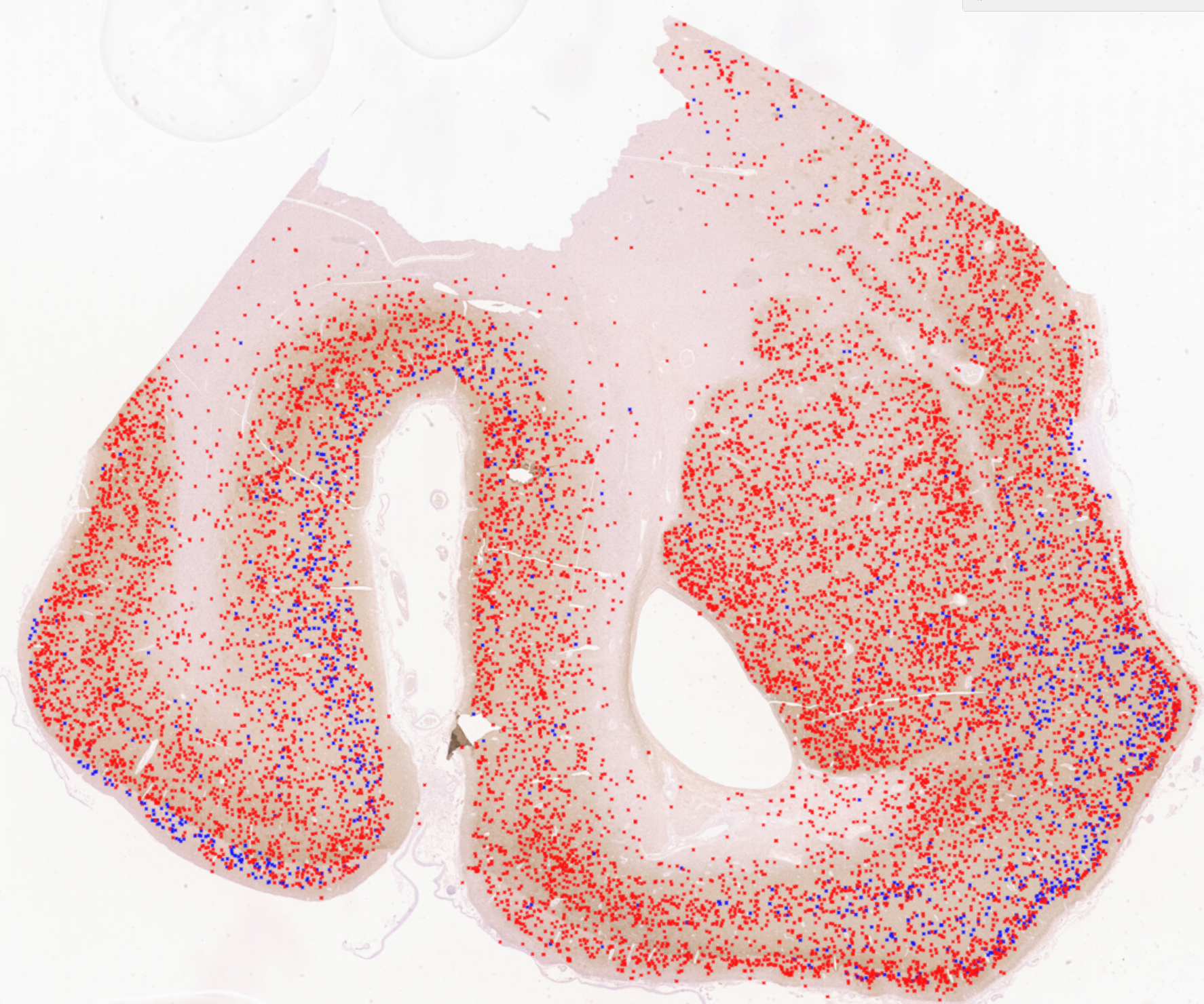
**

**
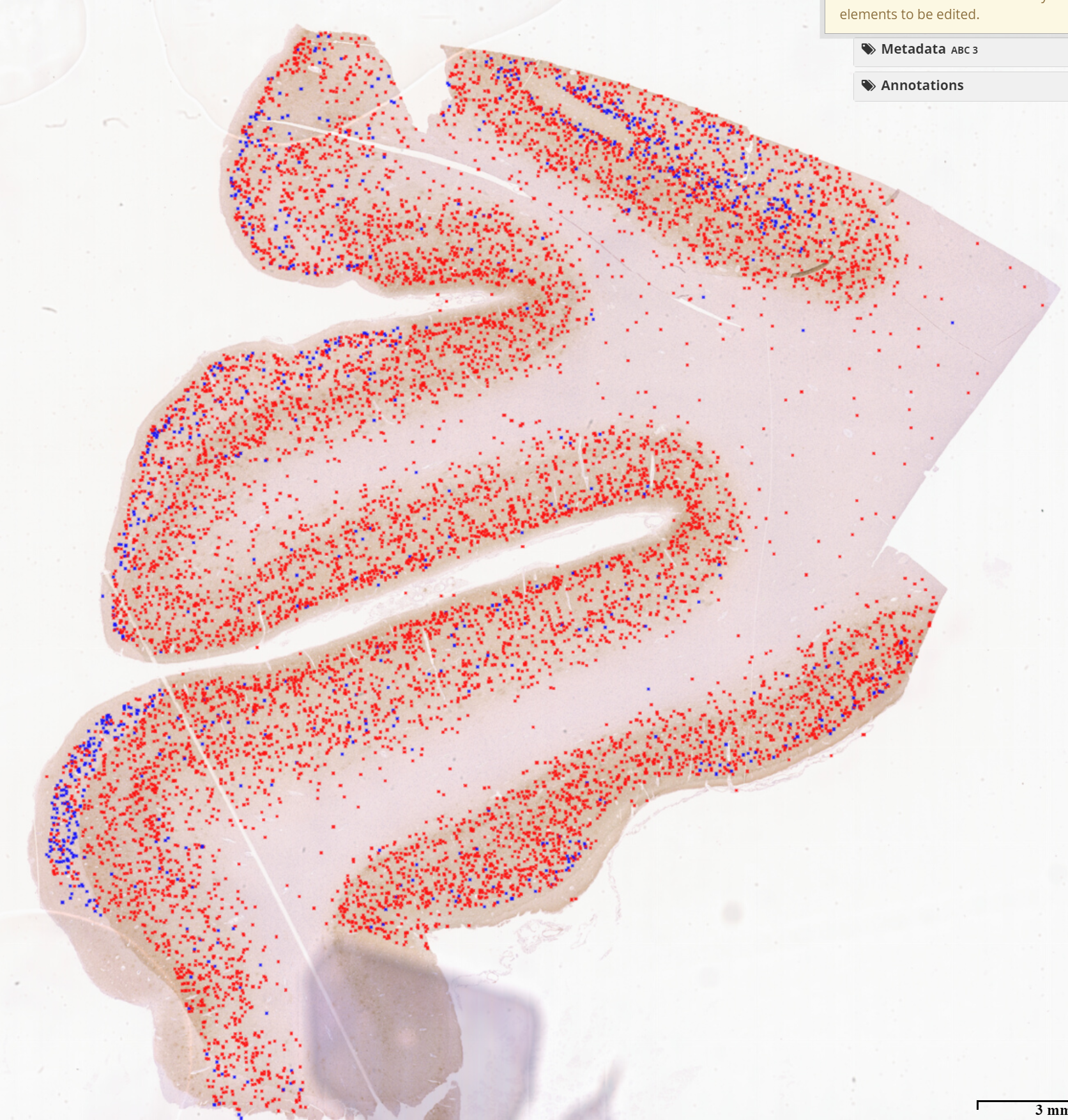

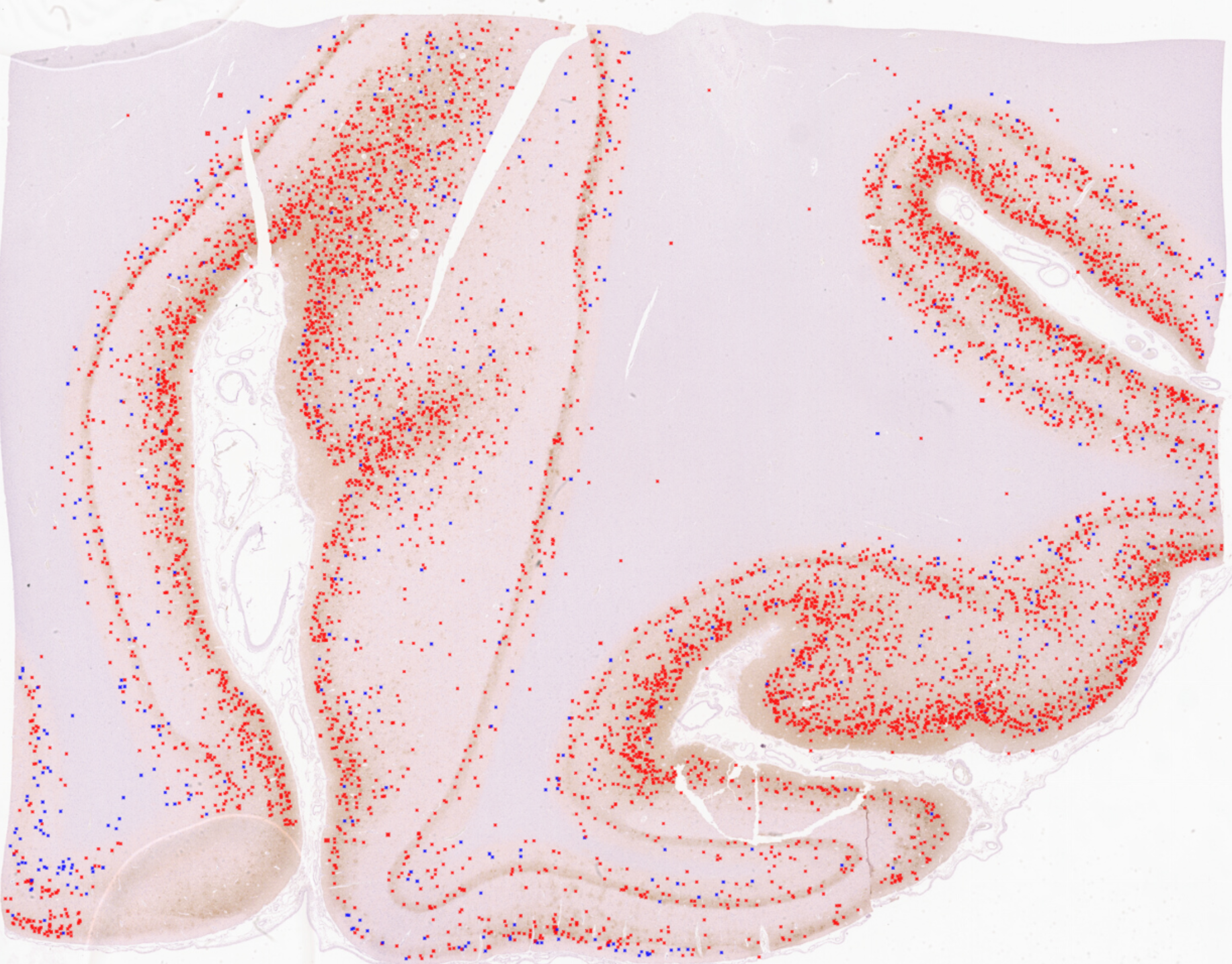
**

**UC Davis Case**


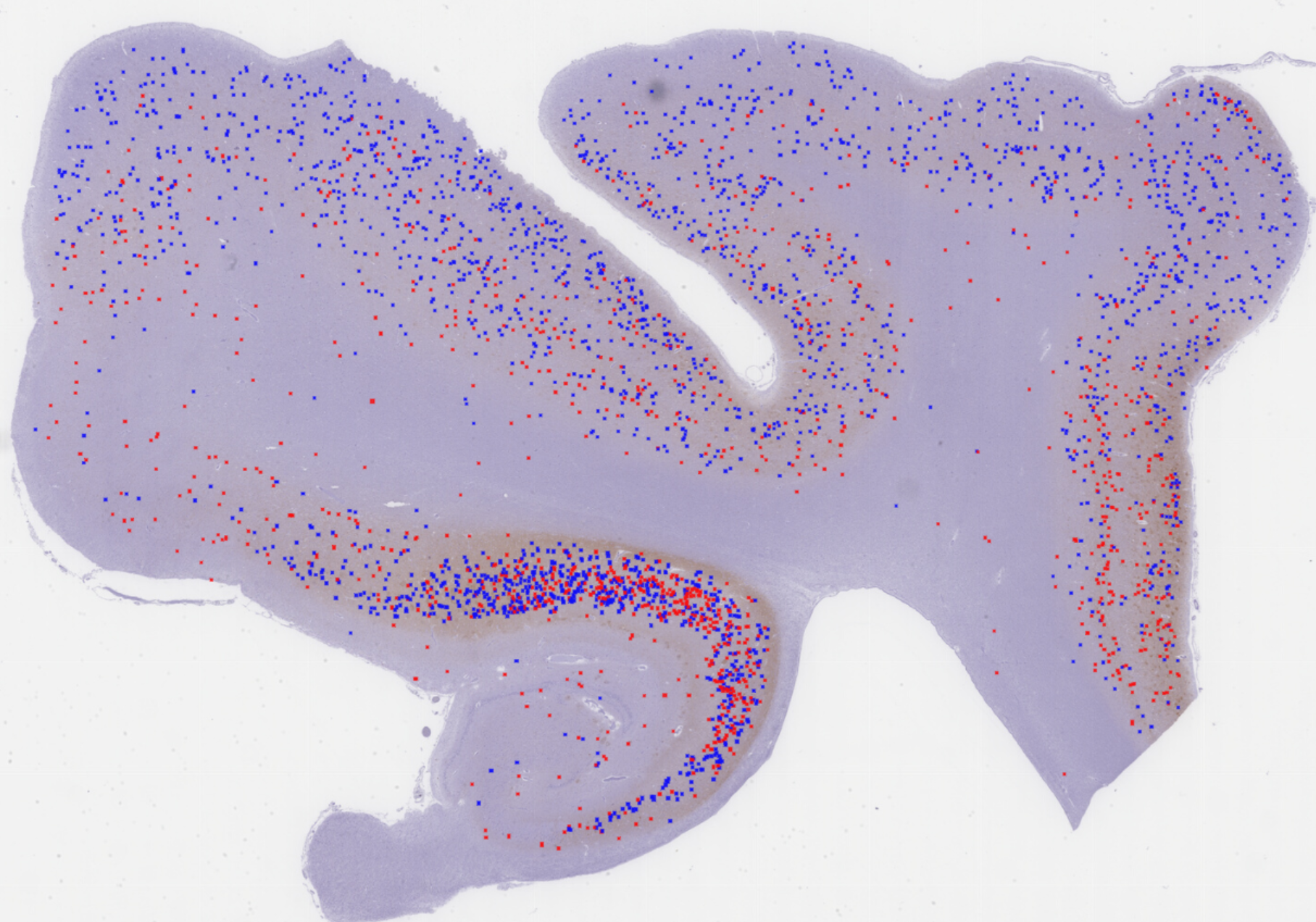

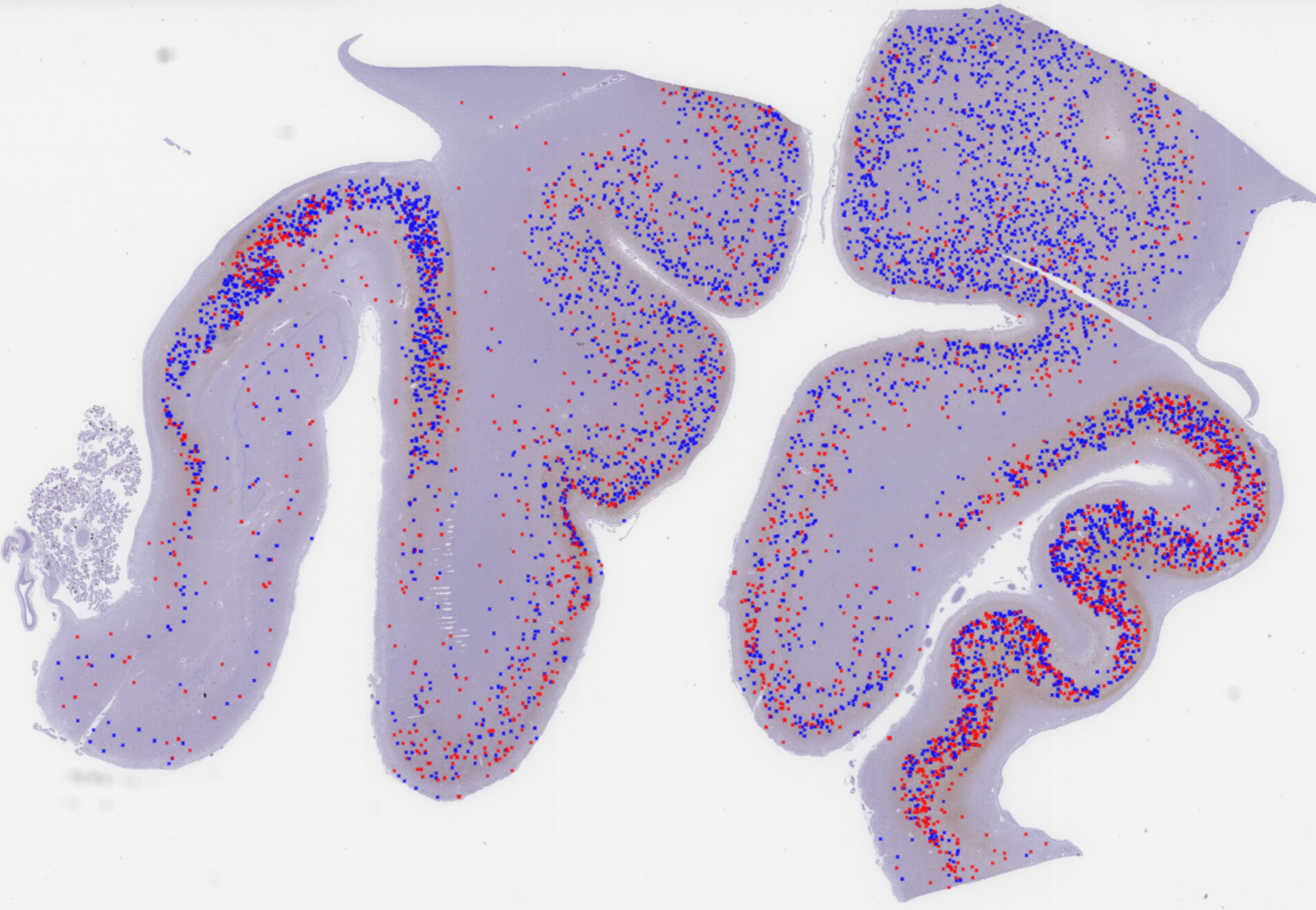


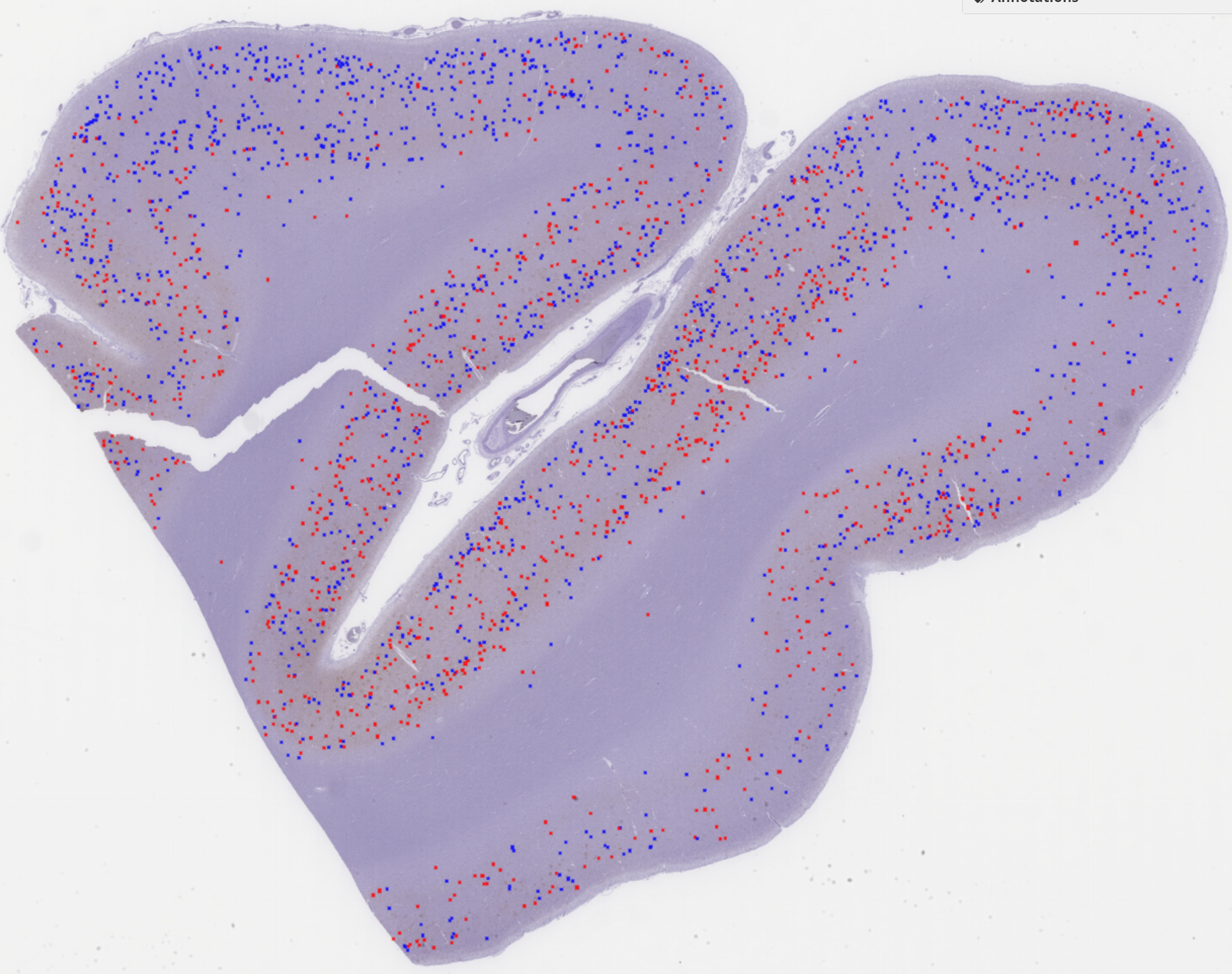

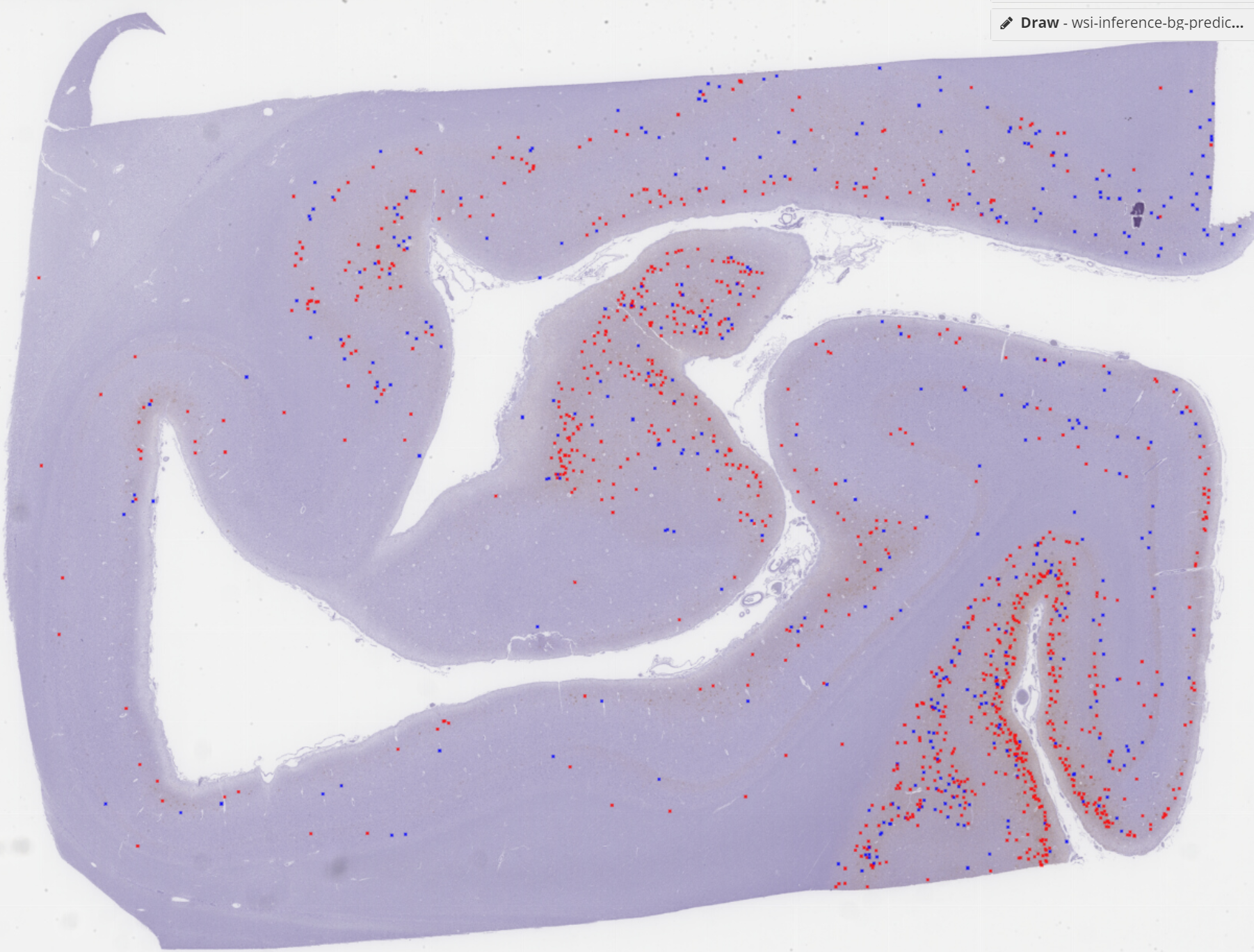


**Supplementary Fig. S11.** **Comparison of Pre-NFT (blue dots) / iNFT (red dots) predictions on an Emory case and UC Davis case of similar pathology (e.g. Braak NFT stage and diagnosis).** These are two cases with Braak NFT stage VI and high AD pathology. Top left images are posterior hippocampus, top right are amygdala (Emory) and anterior hippocampus (UC Davis), bottom left are (temporal cortex) and bottom right are (occipital cortex).


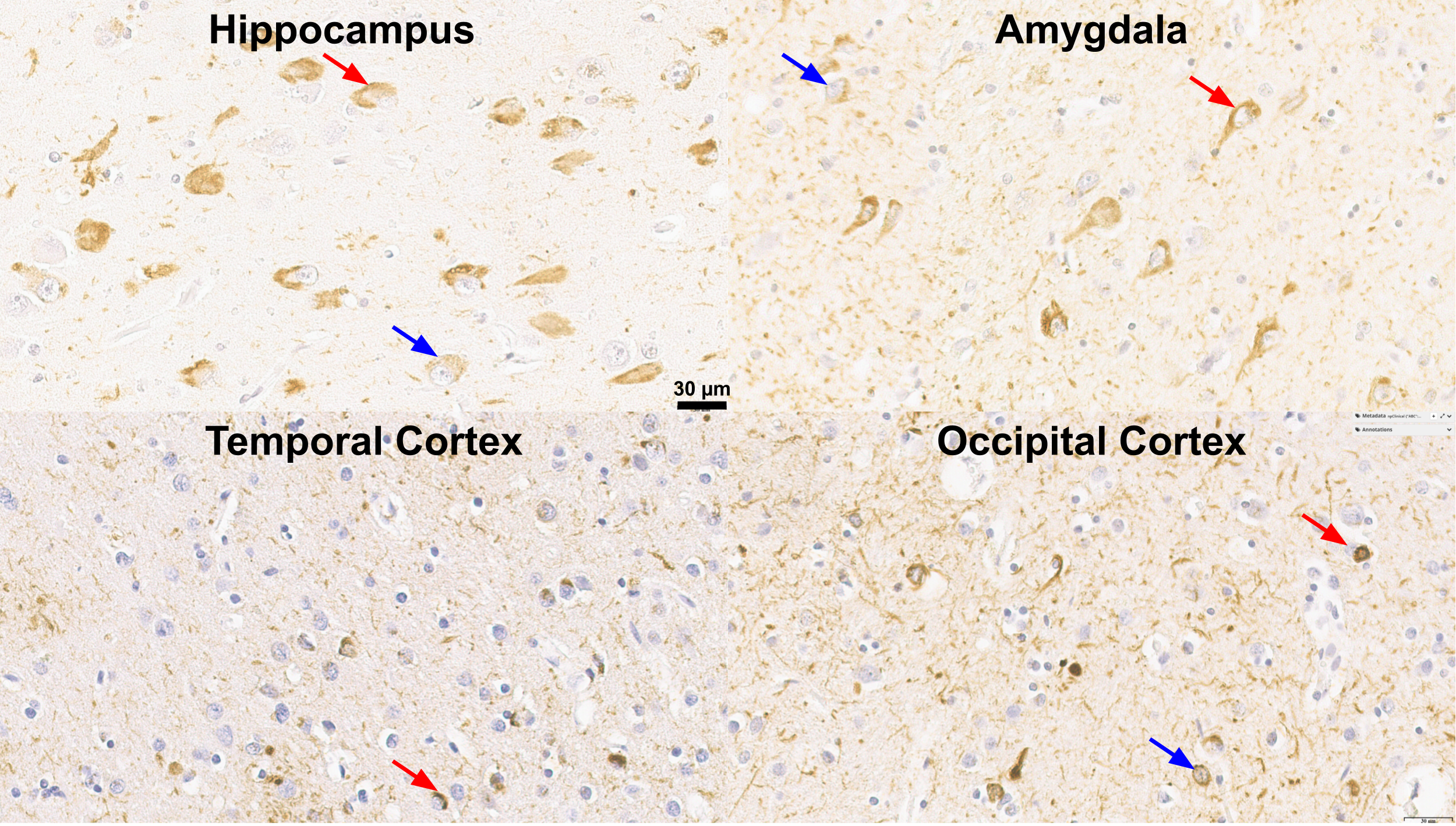


Supplementary Fig. S12. Examples of NFTs in each of the four brain regions from the Emory cohort. Red arrows are examples of iNFTs and blue errors are examples of Pre-NFTs.

**Supplementary Table S1.** Counts of Pre-NFT / iNFT annotations collected

| **Annotator** | **Pre-NFTs** | **iNFT** | **ROIs** | **WSIs** | **Cases** |
| --- | --- | --- | --- | --- | --- |
| Novice 1 | 263 | 1843 | 51 | 43 | 28 |
| Novice 2 | 488 | 1942 | 50 | 41 | 26 |
| Novice 3 | 643 | 2535 | 49 | 36 | 27 |
| Expert 1 | 362 | 2034 | 47 | 35 | 25 |
| Expert 2 | 482 | 1176 | 51 | 40 | 27 |
| Expert 3 | 293 | 2195 | 48 | 41 | 26 |
| Expert 4 | 413 | 1946 | 49 | 33 | 26 |
| Expert 5 | 298 | 1003 | 50 | 37 | 25 |

Summary of annotations collected for each annotator. 15 of the ROIs were the same for each annotator, the rest were uniquely annotated by specific annotators.

**Supplementary Table S2.** Emory-Holdout ROI dataset for testing YOLO model performance.

|  | **Case** | **WSI Filename** | **Braak Stage** | **Brain**  **Region** | **Width**  **(µm)** | **Height**  **(µm)** | **Primary Neuropathology Diagnosis** |
| --- | --- | --- | --- | --- | --- | --- | --- |
| 1 | E19-35 | E19-35_1_TAU.svs | 6 | Posterior hippocampus | 1253 | 676 | AD |
| 2 | E19-70 | E19-70_1_TAU.svs | 3 | Posterior hippocampus | 1245 | 676 | ALS (C9 EXPANSION) |
| 3 | E19-139 | E19-139_6_tau.svs | 1 | Occipital cortex | 680 | 1246 | FTLD-TDP |
| 4 | E19-141 | E19-141_6_tau.svs | 6 | Occipital cortex | 673 | 1246 | AD |
| 5 | E08-145 | E08-145_2A_TAU.svs | 0 | Posterior  hippocampus | 1251 | 678 | CONTROL |
| 6 | E18-45 | E18-45_1_TAU.svs | 4 | Posterior hippocampus | 660 | 1236 | MCI |
| 7 | E18-27 | E18-27_1_TAU.svs | 5 | Posterior  hippocampus | 1252 | 666 | AD |
| 8 | E18-37 | E18-37_2_TAU.svs | 4 | Amygdala | 658 | 1222 | ALS (C9 EXPANSION) |
| 9 | E09-91 | E09-91_1F_tau.ndpi | 2 | Occipital cortex | 1124 | 618 | FTLD-TDP |
| 10 | E10-129 | E10-129_1D_AT8-drop.ndpi | 0 | Temporal cortex | 614 | 1135 | ALS |
| 11 | E10-56 | E10-56_1B_tau.ndpi | 5 | Amygdala | 602 | 1133 | AD |
| 12 | E10-48 | E10-48_1D_tau.ndpi | 4 | Temporal cortex | 611 | 1132 | FTLD-TAU (PSP) |
| 13 | E20-18 | E20-18_4_TAU.svs | 1 | Temporal cortex | 681 | 1252 | CONTROL |
| 14 | E20-71 | E20-71_2 TAU.svs | 3 | Amygdala | 680 | 1253 | ALS (C9 EXPANSION) |
| 15 | E20-72 | E20-72_4 TAU.svs | 3 | Temporal cortex | 682 | 1252 | ALS (C9 EXPANSION) |
| 16 | E20-69 | E20-69_6_TAU.svs | 0 | Occipital cortex | 678 | 1253 | ALS (FUS MUTATION) |
| 17 | E21-84 | A21-84_TAU_2.svs | 0 | Amygdala | 697 | 1280 | ADULT ONSET LEUKOENCEPHALOPATHY WITH AXONAL SPHEROIDS |
| 18 | E21-99 | E21-99_TAU_6.svs | 4 | Occipital cortex | 674 | 1252 | CONTROL |
| 19 | E21-17 | A21-17_4_TAU.svs | 6 | Temporal cortex | 1251 | 679 | AD |
| 20 | E21-24 | A21-24_6_TAU.svs | 3 | Occipital cortex | 680 | 1254 | LBD-NEOCORTICAL |
| 21 | E21-29 | A21-29_4_TAU.svs | 2 | Temporal cortex | 676 | 1245 | VASCULAR DEMENTIA |
| 22 | E17-54 | E17-54_2_tau.svs | 6 | Amygdala | 677 | 1256 | AD |
| 23 | E16-114 | E16-114_4_tau.svs | 5 | Temporal cortex | 1292 | 688 | AD |
| 24 | E16-16 | E16-16_2_tau.svs | 1 | Amygdala | 691 | 1274 | FTLD-TDP (GRN MUTATION) |
| 25 | E16-123 | E16-123_6_tau.svs | 5 | Occipital cortex | 1249 | 682 | AD |
| 26 | E15-125 | E15-125_2_tau.svs | 2 | Amygdala | 1253 | 680 | MICROINFARCTS-PUTAMEN,SUBACUTE;PR,ACUTE |
| 27 | E05-74 | E05-74_2A_TAU.svs | 1 | Posterior  hippocampus | 1272 | 688 | CONTROL |
| 28 | E05-81 | E05-81_1A_TAU.svs | 2 | Posterior  hippocampus | 682 | 1254 | LBD-NEOCORTICAL |

The set of 28 ROIs were annotated by consensus of an expert and a novice, both with knowledge of the project protocol. The dataset was selected to include exactly one ROI from a case of Braak stage 0 to VI and from each of the four brain regions. This dataset was used exclusively to test for performance of the YOLO NFT detection models, to measure the effectiveness of models for identifying boundaries on NFTs and correctly labeling those as either Pre-NFTs or iNFTs.

**Supplementary Table S3.** I**maging Features.**

| # | Feature Name | Used in random forest classifiers? |
| --- | --- | --- |
| 1 | Pre-NFT density (Hippocampus) | Yes |
| 2 | iNFT density (Hippocampus) | Yes |
| 3 | iNFT FOV count (Hippocampus) | Yes |
| 4 | Pre-NFT density (Amygdala) | Yes |
| 5 | iNFT density (Amygdala) | Yes |
| 6 | iNFT FOV count (Amygdala) | Yes |
| 7 | Pre-NFT density (Temporal cortex) | Yes |
| 8 | iNFT density (Temporal cortex) | Yes |
| 9 | Pre-NFT FOV count (Temporal cortex) | Yes |
| 10 | iNFT FOV count (Temporal cortex) | Yes |
| 11 | iNFT Clustering Coef (r=150, Temporal cortex) | Yes |
| 12 | iNFT Clustering Coef (r=200, Temporal cortex) | Yes |
| 13 | iNFT Clustering Coef (r=250, Temporal cortex) | Yes |
| 14 | iNFT Clustering Coef (r=300, Temporal cortex) | Yes |
| 15 | iNFT Clustering Coef (r=350, Temporal cortex) | Yes |
| 16 | iNFT Clustering Coef (r=450, Temporal cortex) | Yes |
| 17 | Pre-NFT density (Occipital cortex) | Yes |
| 18 | iNFT density (Occipital cortex) | Yes |
| 19 | iNFT FOV count (Occipital cortex) | Yes |
| 20 | iNFT Clustering Coef (r=200, Occipital cortex) | Yes |
| 21 | Pre-NFT FOV count (Hippocampus) | No |
| 22 | Pre-NFT Clustering Coef (r=150, Hippocampus) | No |
| 23 | Pre-NFT Clustering Coef (r=200, Hippocampus) | No |
| 24 | Pre-NFT Clustering Coef (r=250, Hippocampus) | No |
| 25 | Pre-NFT Clustering Coef (r=300, Hippocampus) | No |
| 26 | Pre-NFT Clustering Coef (r=350, Hippocampus) | No |
| 27 | Pre-NFT Clustering Coef (r=400, Hippocampus) | No |
| 28 | Pre-NFT Clustering Coef (r=450, Hippocampus) | No |
| 29 | Pre-NFT Clustering Coef (r=500, Hippocampus) | No |
| 30 | Pre-NFT Clustering Coef (r=550, Hippocampus) | No |
| 31 | iNFT Clustering Coef (r=150, Hippocampus) | No |
| 32 | iNFT Clustering Coef (r=200, Hippocampus) | No |
| 33 | iNFT Clustering Coef (r=250, Hippocampus) | No |
| 34 | iNFT Clustering Coef (r=300, Hippocampus) | No |
| 35 | iNFT Clustering Coef (r=350, Hippocampus) | No |
| 36 | iNFT Clustering Coef (r=400, Hippocampus) | No |
| 37 | iNFT Clustering Coef (r=450, Hippocampus) | No |
| 38 | iNFT Clustering Coef (r=500, Hippocampus) | No |
| 39 | iNFT Clustering Coef (r=550, Hippocampus) | No |
| 40 | Pre-NFT FOV count (Amygdala) | No |
| 41 | Pre-NFT Clustering Coef (r=150, Amygdala) | No |
| 42 | Pre-NFT Clustering Coef (r=200, Amygdala) | No |
| 43 | Pre-NFT Clustering Coef (r=250, Amygdala) | No |
| 44 | Pre-NFT Clustering Coef (r=300, Amygdala) | No |
| 45 | Pre-NFT Clustering Coef (r=350, Amygdala) | No |
| 46 | Pre-NFT Clustering Coef (r=400, Amygdala) | No |
| 47 | Pre-NFT Clustering Coef (r=450, Amygdala) | No |
| 48 | Pre-NFT Clustering Coef (r=500, Amygdala) | No |
| 49 | Pre-NFT Clustering Coef (r=550, Amygdala) | No |
| 50 | iNFT Clustering Coef (r=150, Amygdala) | No |
| 51 | iNFT Clustering Coef (r=200, Amygdala) | No |
| 52 | iNFT Clustering Coef (r=250, Amygdala) | No |
| 53 | iNFT Clustering Coef (r=300, Amygdala) | No |
| 54 | iNFT Clustering Coef (r=350, Amygdala) | No |
| 55 | iNFT Clustering Coef (r=400, Amygdala) | No |
| 56 | iNFT Clustering Coef (r=450, Amygdala) | No |
| 57 | iNFT Clustering Coef (r=500, Amygdala) | No |
| 58 | iNFT Clustering Coef (r=550, Amygdala) | No |
| 59 | Pre-NFT Clustering Coef (r=150, Temporal cortex) | No |
| 60 | Pre-NFT Clustering Coef (r=200, Temporal cortex) | No |
| 61 | Pre-NFT Clustering Coef (r=250, Temporal cortex) | No |
| 62 | Pre-NFT Clustering Coef (r=300, Temporal cortex) | No |
| 63 | Pre-NFT Clustering Coef (r=350, Temporal cortex) | No |
| 64 | Pre-NFT Clustering Coef (r=400, Temporal cortex) | No |
| 65 | Pre-NFT Clustering Coef (r=450, Temporal cortex) | No |
| 66 | Pre-NFT Clustering Coef (r=500, Temporal cortex) | No |
| 67 | Pre-NFT Clustering Coef (r=550, Temporal cortex) | No |
| 68 | iNFT Clustering Coef (r=400, Temporal cortex) | No |
| 69 | iNFT Clustering Coef (r=500, Temporal cortex) | No |
| 70 | iNFT Clustering Coef (r=550, Temporal cortex) | No |
| 71 | Pre-NFT FOV count (Occipital cortex) | No |
| 72 | Pre-NFT Clustering Coef (r=150, Occipital cortex) | No |
| 73 | Pre-NFT Clustering Coef (r=200, Occipital cortex) | No |
| 74 | Pre-NFT Clustering Coef (r=250, Occipital cortex) | No |
| 75 | Pre-NFT Clustering Coef (r=300, Occipital cortex) | No |
| 76 | Pre-NFT Clustering Coef (r=350, Occipital cortex) | No |
| 77 | Pre-NFT Clustering Coef (r=400, Occipital cortex) | No |
| 78 | Pre-NFT Clustering Coef (r=450, Occipital cortex) | No |
| 79 | Pre-NFT Clustering Coef (r=500, Occipital cortex) | No |
| 80 | Pre-NFT Clustering Coef (r=550, Occipital cortex) | No |
| 81 | iNFT Clustering Coef (r=150, Occipital cortex) | No |
| 82 | iNFT Clustering Coef (r=250, Occipital cortex) | No |
| 83 | iNFT Clustering Coef (r=300, Occipital cortex) | No |
| 84 | iNFT Clustering Coef (r=350, Occipital cortex) | No |
| 85 | iNFT Clustering Coef (r=400, Occipital cortex) | No |
| 86 | iNFT Clustering Coef (r=450, Occipital cortex) | No |
| 87 | iNFT Clustering Coef (r=500, Occipital cortex) | No |
| 88 | iNFT Clustering Coef (r=550, Occipital cortex) | No |

Density refers to the total number of Pre-NFTs / iNFTs in a squared micron. FOV highest count of Pre-NFTs / iNFTs in a region of size 4 mm^2^. (Average) Clustering coefficient is calculated by building a graph from the center location of Pre-NFTs / iNFTs, connecting nodes if they are within a specified radius (r value). This coefficient is calculated on the FOV chosen with the highest count. Only the top 20 features were used for random forest classification, chosen using recursive feature elimination.

Supplementary Table S4. Annotation Counts by Region

|  | Hippocampus | | Amygdala | | Temporal cortex | | Occipital cortex | |
| --- | --- | --- | --- | --- | --- | --- | --- | --- |
|  | Pre-NFT | iNFT | Pre-NFT | iNFT | Pre-NFT | iNFT | Pre-NFT | iNFT |
| expert1 | 275 | 1470 | 46 | 203 | 16 | 297 | 25 | 64 |
| expert2 | 325 | 827 | 131 | 199 | 21 | 111 | 5 | 39 |
| expert3 | 259 | 1469 | 33 | 331 | 1 | 297 | 0 | 98 |
| expert4 | 371 | 1670 | 14 | 96 | 23 | 148 | 5 | 32 |
| expert5 | 217 | 688 | 60 | 224 | 3 | 47 | 18 | 44 |
| novice1 | 204 | 1207 | 34 | 345 | 24 | 210 | 1 | 81 |
| novice2 | 340 | 1351 | 127 | 427 | 13 | 135 | 8 | 29 |
| novice3 | 457 | 1719 | 133 | 556 | 39 | 231 | 14 | 29 |
| novice4 | 449 | 592 | 17 | 24 | 0 | 0 | 0 | 0 |

Counts of annotations (Pre-NFT & iNFTs) collected from novices and experts by brain region. Novice 4 only annotated 15 ROIs from the hippocampus and amygdala.
